# Predictors of weaning failure in ventilated intensive care patients: a systematic evidence map

**DOI:** 10.1186/s13054-024-05135-3

**Published:** 2024-11-12

**Authors:** Fritz Sterr, Michael Reintke, Lydia Bauernfeind, Volkan Senyol, Christian Rester, Sabine Metzing, Rebecca Palm

**Affiliations:** 1 Faculty of Health, School of Nursing Sciences, Witten/Herdecke University, Alfred-Herrhausen-Straße 50, 58455 Witten, Germany; 2 Faculty of Applied Healthcare Sciences, Deggendorf Institute of Technology, Deggendorf, Germany; 3 Department for Anesthesiology, Intensive Care Medicine, Emergency Medicine and Pain Therapy, Klinikum Landshut, Landshut, Germany; 4 Faculty of Nursing Science and Practice, Paracelsus Medical University Salzburg, Salzburg, Austria; 5 Department of Health Services Research, School VI Medicine and Health Sciences, Carl Von Ossietzky Universität Oldenburg, Oldenburg, Germany; 6 Medical Intensive Care Unit, Klinikum Landshut, Landshut, Germany

**Keywords:** Evidence map, Extubation failure, Intensive care unit, Mechanical ventilation, Predictor, Review, SBT failure, Weaning failure

## Abstract

**Background:**

Ventilator weaning is of great importance for intensive care patients in order to avoid complications caused by prolonged ventilation. However, not all patients succeed in weaning immediately. Their spontaneous breathing may be insufficient, resulting in extubation failure and the subsequent need for reintubation. To identify patients at high risk for weaning failure, a variety of potential predictors has already been examined in individual studies and meta-analyses over the last decades. However, an overview of all the predictors investigated is missing.

**Aim:**

To provide an overview of empirically investigated predictors for weaning failure.

**Methods:**

A systematic evidence map was developed. To this end, we conducted a systematic search in the Medline, Cochrane, and CINAHL databases in December 2023 and added a citation search and a manual search in June 2024. Studies on predictors for weaning failure in adults ventilated in the intensive care unit were included. Studies on children, outpatients, non-invasive ventilation, or explanatory factors of weaning failure were excluded. Two reviewers performed the screening and data extraction independently. Data synthesis followed an inductive approach in which the predictors were thematically analyzed, sorted, and clustered.

**Results:**

Of the 1388 records obtained, 140 studies were included in the analysis. The 112 prospective and 28 retrospective studies investigated a total of 145 predictors. These were assigned to the four central clusters ‘Imaging procedures’ (n = 22), ‘Physiological parameters’ (n = 61), ‘Scores and indices’ (n = 53), and ‘Machine learning models’ (n = 9). The most frequently investigated predictors are the rapid shallow breathing index, the diaphragm thickening fraction, the respiratory rate, the P/F ratio, and the diaphragm excursion.

**Conclusion:**

Predictors for weaning failure are widely researched. To date, 145 predictors have been investigated with varying intensity in 140 studies that are in line with the current weaning definition. It is no longer just individual predictors that are investigated, but more comprehensive assessments, indices and machine learning models in the last decade. Future research should be conducted in line with international weaning definitions and further investigate poorly researched predictors.

*Registration, Protocol*: 10.17605/OSF.IO/2KDYU

**Supplementary Information:**

The online version contains supplementary material available at 10.1186/s13054-024-05135-3.

## Background

While the number of mechanically ventilated patients in intensive care units (ICU) is increasing worldwide [1–4], the number of those who cannot be successfully weaned also remains high. Prolonged weaning or death occurs in one of four patients undergoing mechanical ventilation (MV), despite at least one spontaneous breathing trial (SBT) was performed [5]. In addition, patient mortality increases depending on the duration of ventilation [6].

Patients successfully weaned demonstrate a varying rate of extubation failure (EF) depending on the study and the population [7]. However, reintubation of patients is significantly associated with increased ICU and in-hospital mortality [8].

In recent decades, research has focused on weaning failure (WF), which is defined as reintubation or death within seven days [6]. Today, much is known about the pathophysiology of WF [9], with various risk factors (e.g. age, gender, underlying disease, psychological determinants) being investigated in several studies [10–13]. Healthcare professionals caring for ventilated patients in an ICU need to be aware of these factors to prevent possible WF. In this regard, prevention also includes identifying patients at high risk for WF at an early stage.

‘Predictive factors’ or ‘predictors’ indicating such a risk have been investigated in many studies; in individual studies (e.g. [14–16]) as well as meta-analyses (e.g. [17, 18]). What is missing, is an overview of existing studies to map the evidence and to identify possible research gaps with respect to certain predictors. In addition, the underlying definitions of weaning outcomes were adapted in 2007 [19] and in 2017 [6]. Because of this, it can be assumed that not all studies are in line with the current weaning definition [6].

### Aim and research question

Based on this, we aimed to provide an overview of predictors for WF, reconstruct the trends in research over the years and identify potential research gaps. Thereby, we followed the research question: “Which predictors for the failure of ventilator weaning in adult intensive care patients are already empirically investigated?”.

## Methods

We conducted a literature review and designed a systematic evidence map (SEM). This enabled us to present the existing body of knowledge, uncover research activities and trends, map relationships between predictors and outcomes and derive implications for further research. In this regard, we were guided by the results and methodological recommendations of Miake-Lye et al. [20]. Since an SEM does not yet represent a differentiated methodology [21] and is close to scoping reviews [20], we also followed the methodological guidelines for scoping reviews [22, 23]. The reporting of our abstract and main body is guided by the recommendations of the ‘Preferred Reporting Items for Systematic reviews and Meta-Analyses’ (PRISMA) [24] and its extension for scoping reviews (PRISMA-ScR) [25].

### Protocol and registration

According to the methodological guidelines [24, 26], we registered our review and published a research protocol in the Open Science Framework (10.17605/OSF.IO/2KDYU) in February 2024.

### Eligibility criteria

In line with the PCC scheme (Person, Concept, Context) which is recommended for scoping reviews [22, 26], we defined adult patients receiving MV in an ICU as the persons of interest in our review. Patients cared for in long-term care settings or at home, as well as infants or children were excluded. We also excluded patients in weaning centers or step-down units, contrary to our protocol, as these were primarily characterized by hemodynamic stability and prolonged ventilation, which would have resulted in an increased heterogeneity.

Following the aim, we focused on predictors of WF as the concept of interest. We understand *predictive factors* as those aspects to make assumptions about the probability of the occurrence or absence of health risks (in our case: WF). These include various parameters and clinical findings that are collected individually or combined in assessments and then tested in a statistical analysis (usually logistic regression) [27]. In contrast, *explanatory factors* are mainly concerned with causality, or the direction of a disease [27], thereby only making assumptions about the etiology of WF, but not its probability. Based on this differentiation, we only included predictors in our review. Explanatory factors were rigorously excluded.

WF is our context of interest. In detail, we considered SBT failure, EF or decannulation failure (DF) as WF as long as the patients were still receiving respirator support. Extubation or decannulation has failed if the patient requires reintubation/ recannulation or dies within seven days [6]. Studies not reporting on any of these outcomes or not being in line with our underlying definition were consecutively excluded. For example, this is the case when studies declare non-invasive ventilation (NIV) as WF. In contrast to the International Consensus Conference (ICC) classification from 2007 [19], the use of NIV is no longer considered weaning failure since the WIND study in 2017 [6].

In our SEM, we only included German or English original studies and those reviews that reported new findings (e.g. by meta-analysis). According to our definition of predictors, articles had to provide a statistical analysis to calculate probabilities. Analysis of sensitivity and specificity could entail further information, but was not mandatory. Studies were excluded if they only provided information on group comparisons (e.g., by student’s t-test). Grey literature, non-scientific articles, and reviews, which only reported on weaning predictors second-hand, were also excluded.

### Information sources

To answer the underlying research question, we conducted a systematic literature search in the three databases Medline (via PubMed), Cumulative Index to Nursing and Allied Health Literature (CINAHL), and Cochrane Library in December 2023. To identify potentially missed references, we conducted an additional hand search in Google Scholar and LIVIVO in June 2024. We also carried out citation searching [28], using 15 identified reviews as seed references.

### Search strategy

To identify relevant keywords and medical subject headings for our search, two reviewers (FS, MR) conducted an orienting search in Medline and CINAHL independently. Based on this, the systematic search was collaboratively developed by our team. The final search string, its results, and additional searches are depicted in Additional file 1.

### Selection of sources of evidence

After the search results were exported from the databases, we merged them into a common Citavi project to conduct a duplicate scan. Two reviewers (FS, MR) then performed a blinded title, abstract and full-text screening in the Rayyan web application. Arising conflicts were solved in group discussions with two additional reviewers (LB, CR).

### Data charting process

Data from included studies was extracted independently into a predefined table (see protocol) by two reviewers (FS, MR) and compared afterwards. Any conflicts were again discussed and resolved with two additional reviewers (LB, CR).

### Data items

In detail, we extracted information on the authors of the study, its year and country of publication, the study design, population, and setting. Furthermore, the predictive factors, the investigated outcomes, and the results of the studies were extracted (see protocol).

### Synthesis of results

After data from all included studies had been extracted into a Microsoft Excel sheet, we followed an inductive approach to thematically group similar predictors and their related outcomes [23]. After discussing potential overlaps and gaps, we categorized our results into main and subclusters. The application Cytoscape was then used to visualize the identified links between predictors and outcomes in network diagrams [29]. Matplotlib was used to create further diagrams and Figures [30].

## Results

### Selection of sources of evidence

**Fig. 1:**
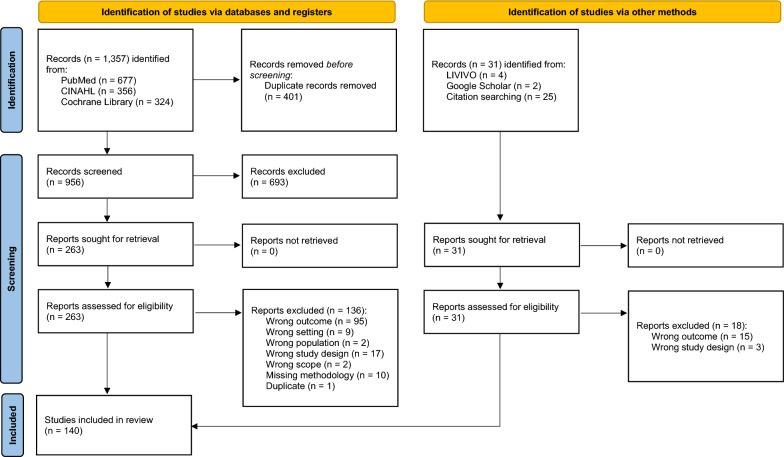
PRISMA flow diagram of the search and screening process

The systematic literature search in the three databases Medline, CINAHL, and Cochrane Library yielded 1357 records. Additional searches and citation searching resulted in a further 31 references. Excluding 401 duplicates, 987 records were screened for their titles and abstracts. After removing another 693 records, 294 articles remained for full-text screening. During this process, 154 studies were excluded (see Additional file 2). Finally, 140 studies met the eligibility criteria and were included in our SEM. The entire search and screening process are illustrated in Fig. 1.

### Characteristics of sources of evidence

**Fig. 2:**
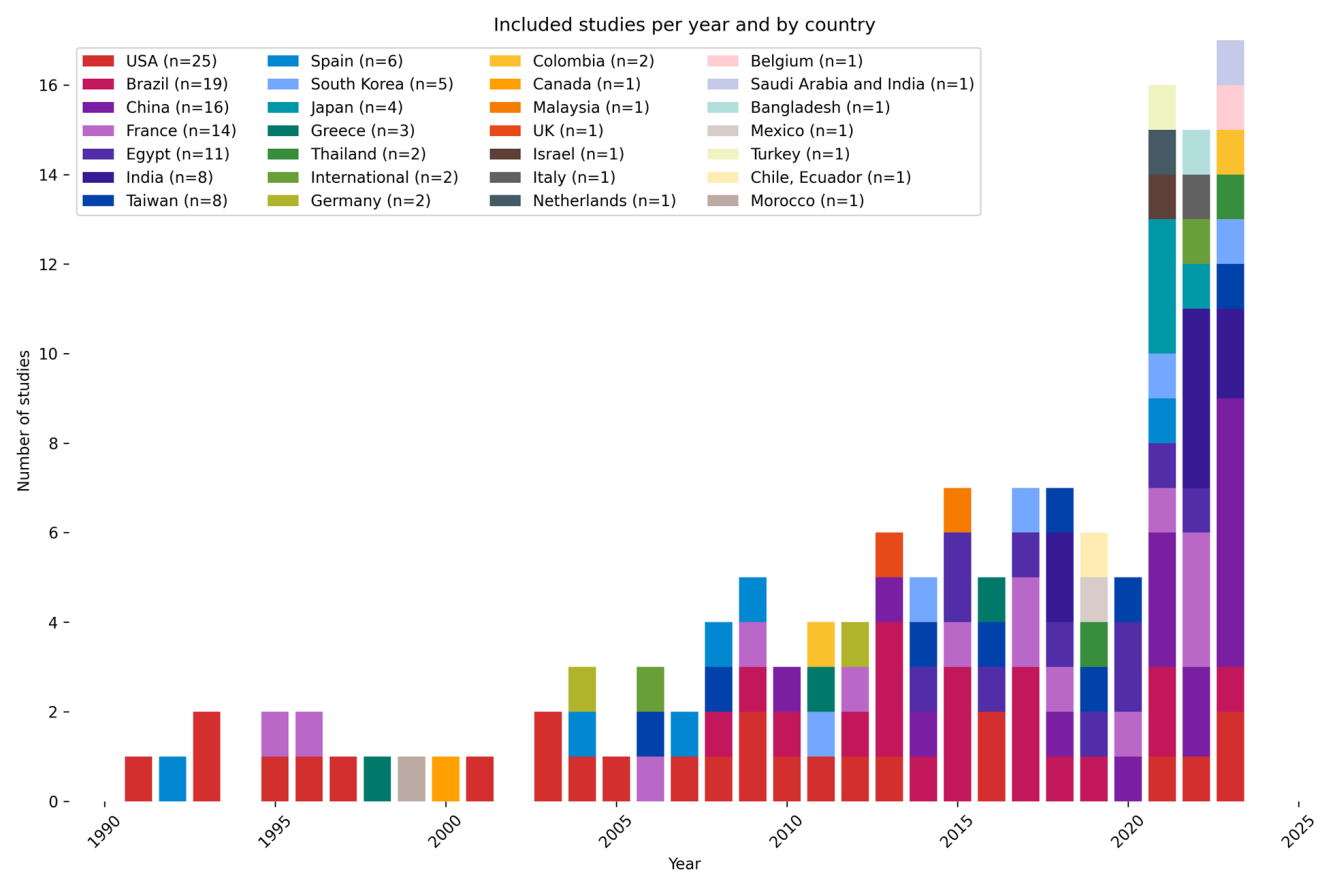
Included studies per year and by country

In this review, we included 112 prospective and 28 retrospective studies. These were published between 1991 and 2023, with a sharp increase in number of studies since 2019. Most studies were conducted in the USA (n = 25), Brazil (n = 19), China (n = 16), France (n = 15), and India (n = 9) (see Fig. 2). The number of patients included ranges from 24 [31] to 6583 [32].

**Fig. 3:**
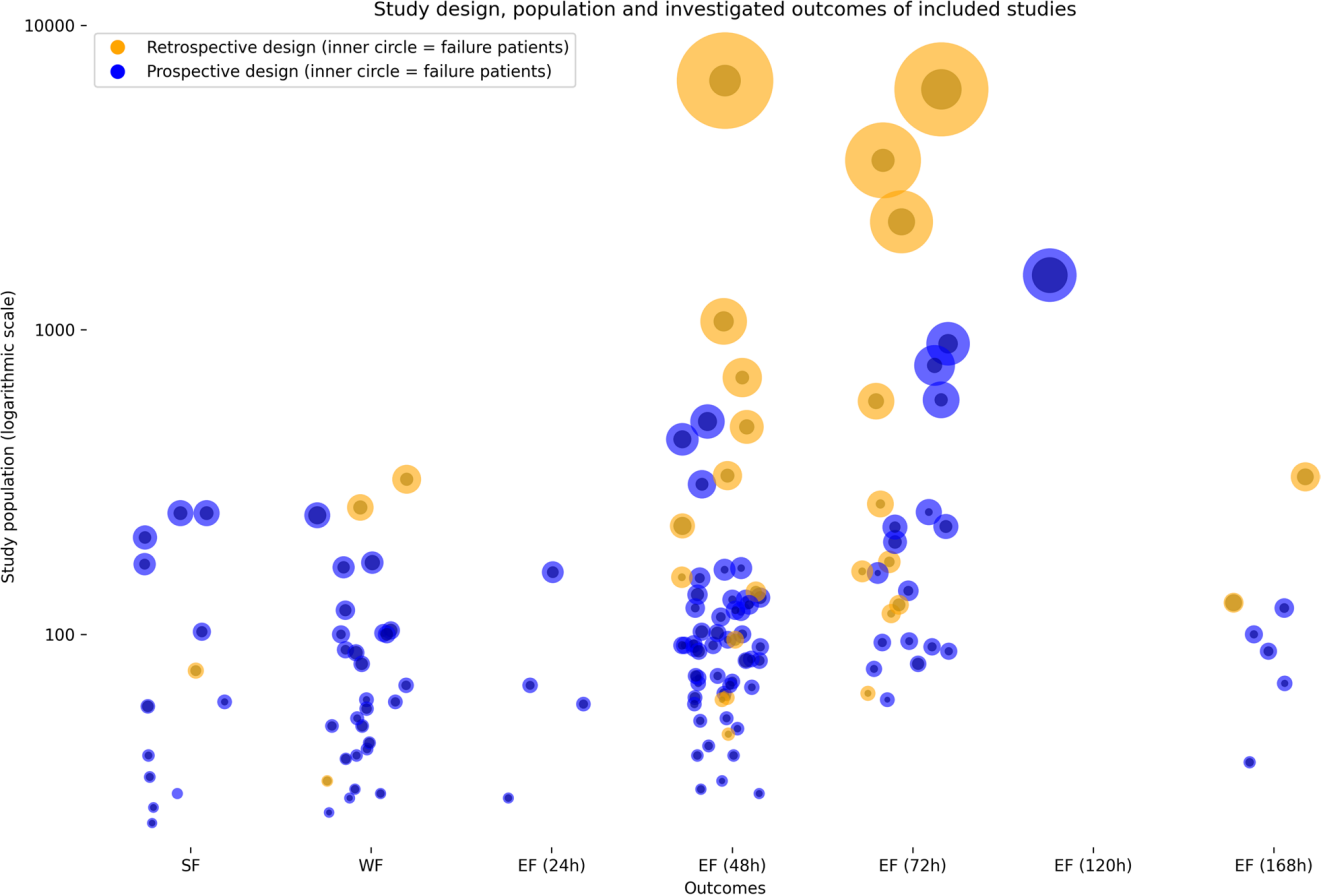
Study design, population and investigated outcomes of included studies

Overall, 13 studies examined predictors for SBT failure, 97 for EF, one study for both EF and SBT failure, and 29 for WF as a combination of EF and SBT failure. The relationship between study design, sample size and outcomes is shown in Fig. 3. The most and the largest studies investigated EF within 48 h or 72 h. Further information on the individual studies is available in the study characteristics table (see Additional file 3).

### Synthesis of results

In the included studies, a total of 145 predictors were identified. These were assigned to the four main clusters ‘Imaging procedures’ (n = 22), ‘Physiological parameters’ (n = 61), ‘Scores and indices’ (n = 53), and ‘Machine learning models’ (n = 9). These main clusters, their subclusters and respective predictors are presented below.

### Cluster 1—imaging procedures

The first main cluster comprises 22 predictors resulting from the visualization of individual or multiple body regions and is further subdivided into five subclusters. The first subcluster ‘Diaphragm ultrasound’ includes the predictors passive cephalic excursion of the diaphragm (PCED) [33], diaphragm excursion (DE) [33–47], diaphragm thickening fraction (DTF) [34–39, 42–46, 48–56], diaphragm peak velocity [33, 35, 40], and diaphragm longitudinal strain (DLS) [43].

The second subcluster ‘Thorax ultrasound’ comprises the predictors B-Lines [49, 52, 57], lung ultrasound score [42, 54], transthoracic echocardiography (TTE) [52, 58–62], transesophageal echocardiography (TEE) [63], and a holistic ultrasound assessment (heart, lung, diaphragm) [52].

The third subcluster ‘Muscle ultrasound’ consists of the predictors thickness of musculus rectus femoris (Trf) [36], thickness of musculus vastus intermedius (Tvi) [36], and the parasternal intercostal thickening fraction (TFic) [51, 64].

The fourth subcluster ‘Ultrasound indices’ comprises predictors that combine ultrasound results with other factors. These include TFic/DTF [51], Trf + Tvi [36], respiratory rate (RR)/DTF [35], RR/DE [35, 37], rapid shallow diaphragmatic index (RSDI = [RR/Tidal volume]/DE) [37], ultrasound diaphragmatic load ([RR * DE^3^]/DTF) [37], and ultrasound respiratory muscle load ([RR * DE^3^]/[DTF + accessory muscle activity]) [37].

The fifth subcluster 'Non-ultrasound imaging' includes predictors resulting from other sources of visualization. These are the electrical impedance tomography [65–67], and the radiographic score (after chest x-ray) [68].

**Fig. 4:**
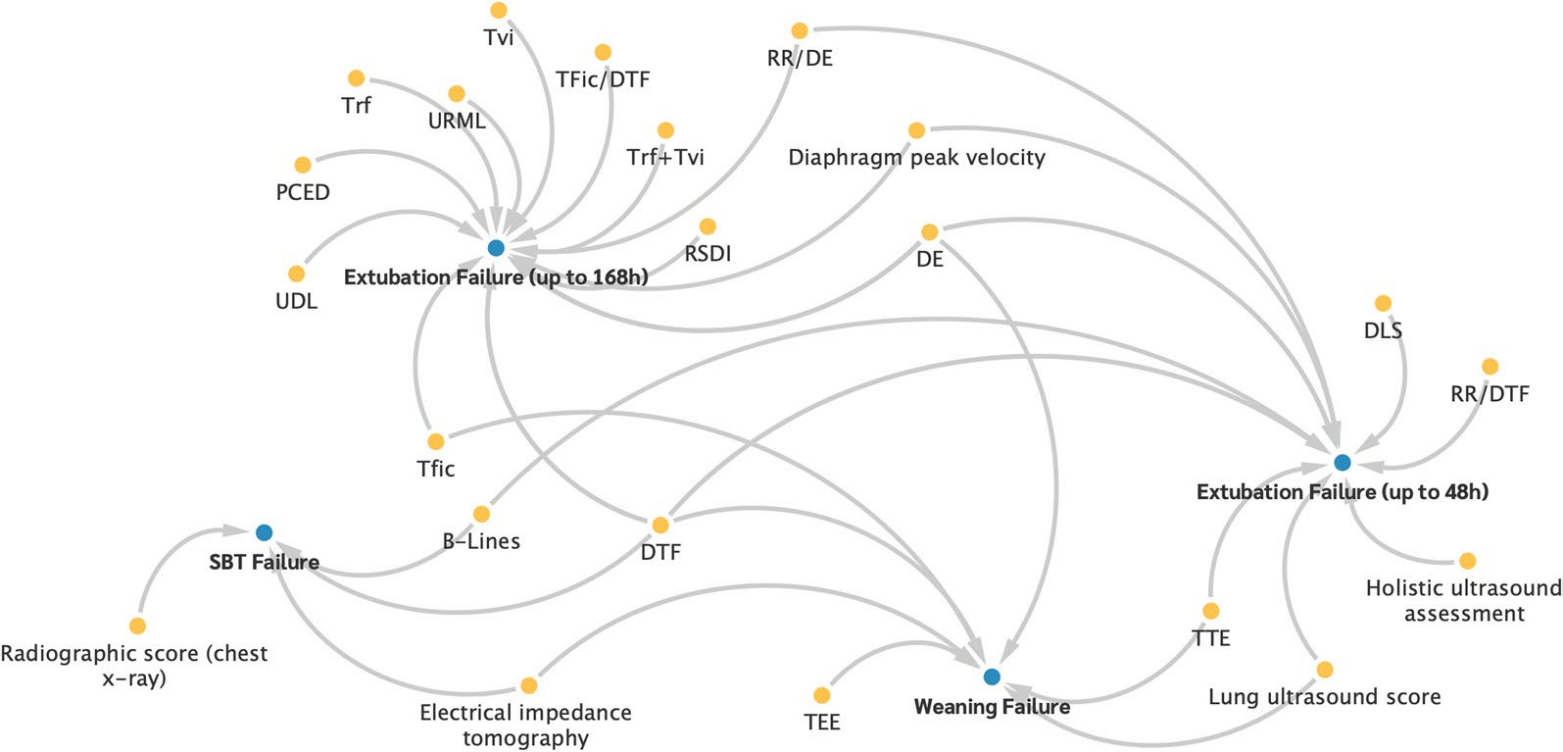
Association of the predictors from cluster 1 with weaning failure outcomes

Figure 4 illustrates the relationship between the predictors in the first cluster ‘Imaging procedures’ and the weaning outcomes. Thereby, the arrows indicate which factors (yellow dots) were examined as predictors in connection with certain outcomes (blue dots).

### Cluster 2—physiological parameters

The second main cluster consists of 61 predictors resulting from the physiology of the patients and is divided into a further four subclusters. The first subcluster ‘Monitoring’ comprises the predictors fluid balance [49, 58, 69–75], mean blood pressure (MBP) [76], central venous pressure (CVP) [77], heart rate (HR) [73, 77–82], heart rate variability (HRV) [83], thoracic fluid content (TFC) [84], amount of secretion [70, 85–90], SBT [90, 91], and cerebral cortex perfusion [31].

The second subcluster ‘Ventilatory parameters’ includes the predictors mean airway pressure (MAP) [71, 92], lung compliance [92–94], tidal volume (Vt) [47, 80, 91, 94–103], minute ventilation (Ve) [47, 91, 94, 96, 98, 100, 101, 104–107], P0.1 [44, 98, 108–110], maximal inspiratory pressure (MIP) [32, 35, 44, 47, 91, 94, 96, 97, 99, 101, 105, 108, 111], maximal expiratory pressure (MEP) [32, 82, 101], functional residual capacity (FRC) [112], cuff leak volume [32, 111], RR [52, 77–81, 94, 96–102, 113–116], vital capacity (VC) [35, 101], work of breathing (WOB) [117], pressure frequency product (PFP) [60, 93], minute ventilation recovery time (VeRT) [104, 106, 118], positive end-expiratory pressure (PEEP) [114], inspiratory pressure (Pi) [96, 100], FiO_2_ [114], mechanical power (MP) [100], and the driving pressure (ΔP) [92].

The third subcluster ‘Laboratory parameters’ consists of the predictors B-type natriuretic peptide (BNP) [58, 93, 107, 114, 119–123], hemoglobin (Hb) [52, 76, 80, 88, 90, 124–126], paO_2_ [78, 99], paCO_2_ [86, 90, 99, 106, 119, 127, 128], gastric intramucosal pH [95, 97], gastric intramucosal pCO_2_ [95], ScvO_2_ [77, 129], HCO_3_^−^ [84, 114], pH [79, 90, 103, 114], serum cholinesterase (SChE) [78], red blood cell acetylcholinesterase (AChE) [130], serum-anion gap [131], delta of gastric and arterial pCO_2_ [98], malondialdehyde (MDA) [132], vitamin C [132], nitric oxide concentration [132], alanine aminotransferase (ALT) [133], albumin [49, 90, 126], mean platelet volume (MPV) [134], leukocyte [52, 134], SaO_2_ [31], bilirubin [114], blood glucose [114], aPTT [114], blood urea nitrogen level (BUN) [124], total proteins [90], creatinine [52], and CRP [52, 134].

The fourth subcluster ‘Muscle strength’ contains the predictors cough effectiveness [70, 85, 88, 89, 135], cough peak flow (CPF) [33, 39, 87, 88, 90, 103, 125, 135–137], handgrip strength [138, 139], tongue protrusion [138], and semi-quantitative cough strength score (SCSS) [140].

**Fig. 5:**
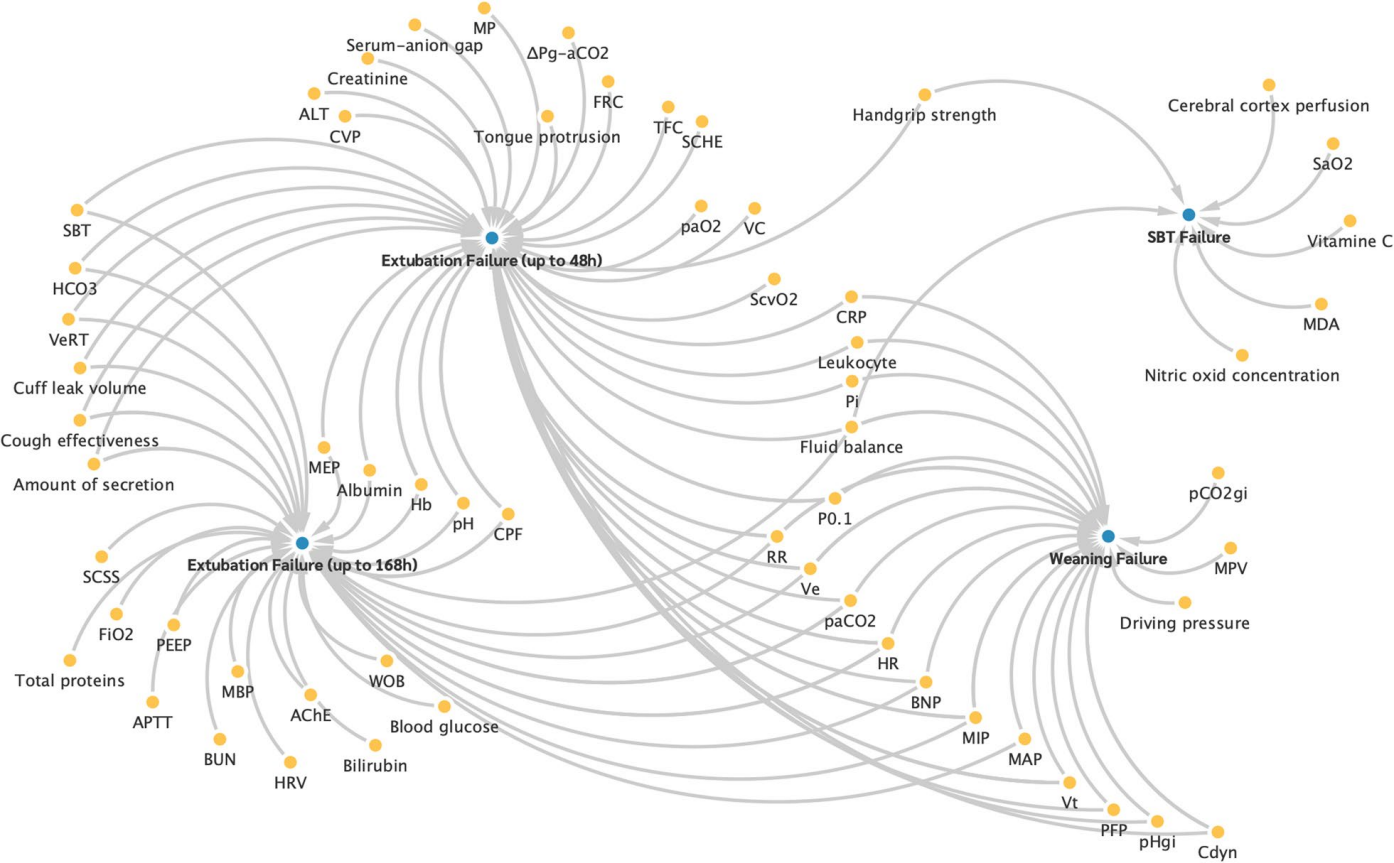
Association of the predictors from cluster 2 with weaning failure outcomes

The connection between the predictors in the second cluster and the weaning outcomes is shown in Fig. 5.

### Cluster 3—scores and indices

The third main cluster consists of 53 predictors combining two or more variables and is divided into four subclusters. The first subcluster ‘Respiratory indices’ includes the P/F ratio [47, 52, 82, 84, 88, 90, 91, 95, 99, 114, 131, 140–144], the rapid shallow breathing index (RSBI) [32, 34, 37, 41, 43, 44, 46, 47, 49, 54, 58–60, 65, 72, 74, 75, 80–82, 84, 88, 90, 91, 94–102, 105–113, 125, 126, 135, 140, 141, 143, 145–154], respiratory system compliance [143], ROX index [76], SpO_2_/FiO_2_ [76], composite score (DTF + RSBI) [48], alveolar-arterial oxygen difference [144], P0.1/MIP [108, 149], timed inspiratory effort index (TIE) [155], twitch tracheal pressure in response to magnetic phrenic stimulation (Ptr,stim) [50], P0.1*RSBI [109, 110], inspiratory effort quotient (IEQ) [149], CROP index [94, 99], tension time index (TTI) [153], systemic DO_2_ [31], Pi/MIP [96], hypercapnic ventilatory response (∆Ve/∆PaCO2) [128], hypercapnic respiratory drive response (∆P0.1/∆PaCO2) [128], ∆P0.1/PaCO2 [127], ∆Ve/PaCO2 [127], RSBI/body weight [152], PaO_2_/PAO_2_ [94], weaning index [105, 156], integrative weaning index (IWI) [157, 158], modified integrative weaning index [159], CPF/secretion model [87], FRC/predicted body weight (pBW) [112], and Vt/ body weight [94].

The second subcluster ‘Disease scores and assessments’ comprises the predictors APACHE II [32, 60, 74, 78, 90, 114, 125, 140, 160], SOFA score [78, 143, 161, 162], lung injury score (LIS) [114], GOCA (gas exchange, organ failure, cause, associated disease) score [114], HACOR score [161], Charlson comorbidity index (CCI) [161], clinical frailty score (CFS) [36], NUTRIC score [49], BMI [62, 111, 143, 152], total body surface are burned (TBSA) [79], MRC muscle strength score [51, 124, 139], therapeutic intervention scoring system (TISS) scale [82], and reintubation scale calculation (RISC) score [71].

The third subcluster ‘Neurologic and bulbar assessments’ contains the predictors Glasgow coma scale (GCS) [32, 71, 80, 85, 86, 111, 126, 137, 140, 163, 164], a self-developed risk score (sex, GCS, secretion, cough, MV) [85], VISAGE (visual pursuit, age, swallowing attempts, GCS) score [39, 164], global swallowing pattern assessment [165], following commands (eyes, hands, tongue) [135], STAGE (swallowing, tongue protrusion, cough, suctioning, motor response) score [166], ENIO score [167], and the respiratory insufficiency scale-intubated (RIS-i) [39].

The fourth subcluster ‘Dyspnoea assessments’ includes the predictors MV-respiratory distress observation scale (MV-RDOS) [148], dyspnoea visual analogue scale (Dyspnoea-VAS) [51], and the intensive care respiratory distress observational scale (IV-RDOS) [51].

**Fig. 6:**
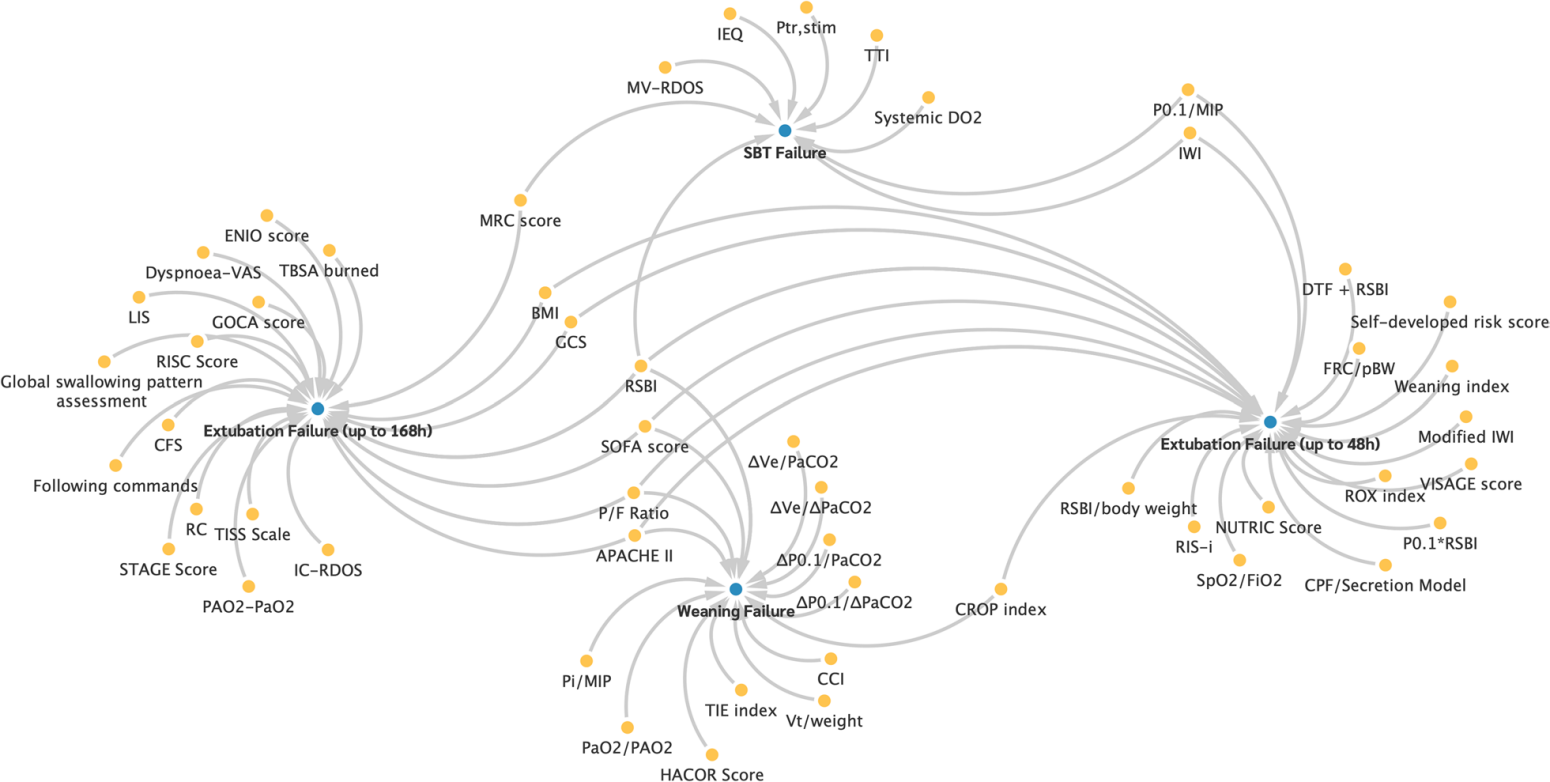
Association of the predictors from cluster 3 with weaning failure outcomes

The connection between the predictors in the third cluster and the weaning outcomes is shown in Fig. 6.

### Cluster 4—machine learning models

**Fig. 7:**
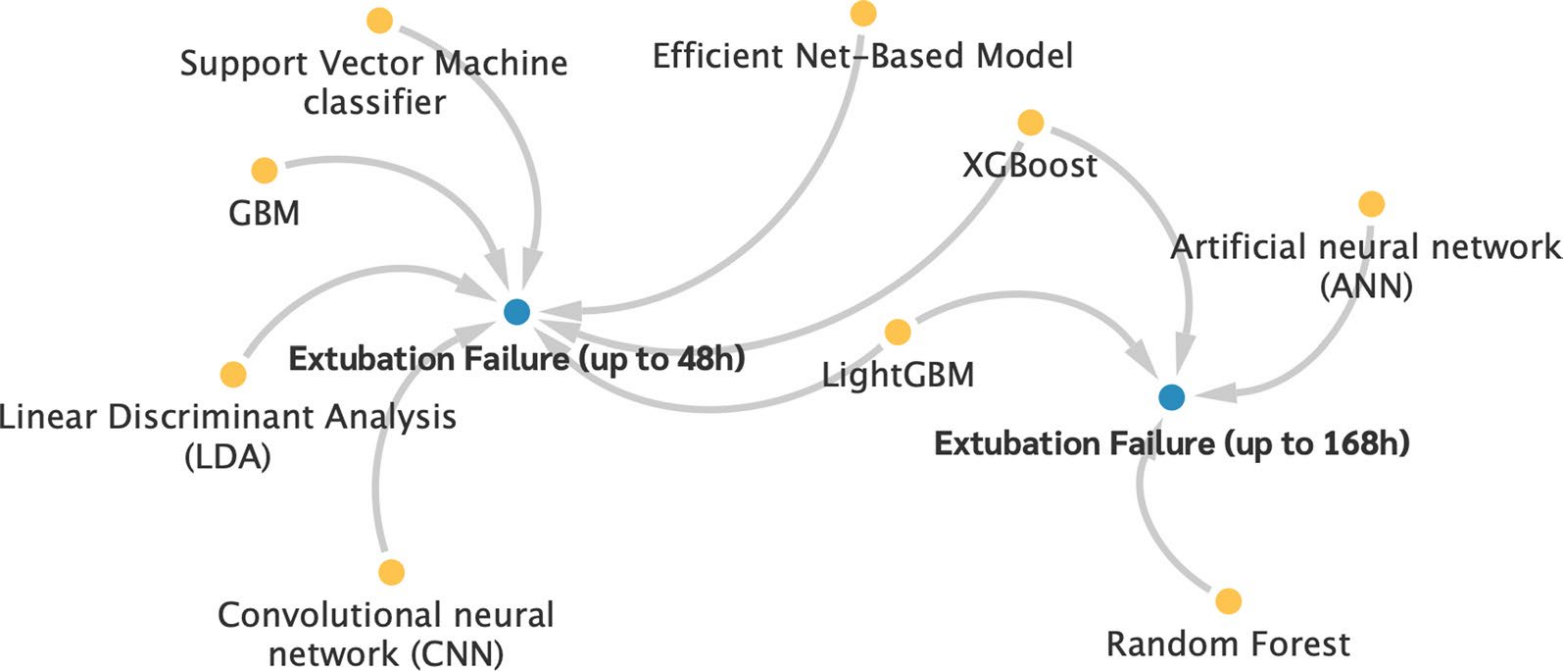
Association of the predictors from cluster 4 with weaning failure outcomes

The fourth main cluster includes a further nine predictors that combine a high number of parameters, values, and indices in machine learning models. These are the Support Vector Machine Classifier [168], LightGBM [169, 170], GBM [168], Linear Discriminant Analysis [168], Random Forest [170], XGBoost [100, 170], Convolutional Neural Network (CNN) [150], Artificial Neural Network (ANN) [82], and Efficient Net-Based Model [169]. The connection between these predictors and the weaning outcomes is shown in Fig. 7.

### Cluster overview

**Fig. 8:**
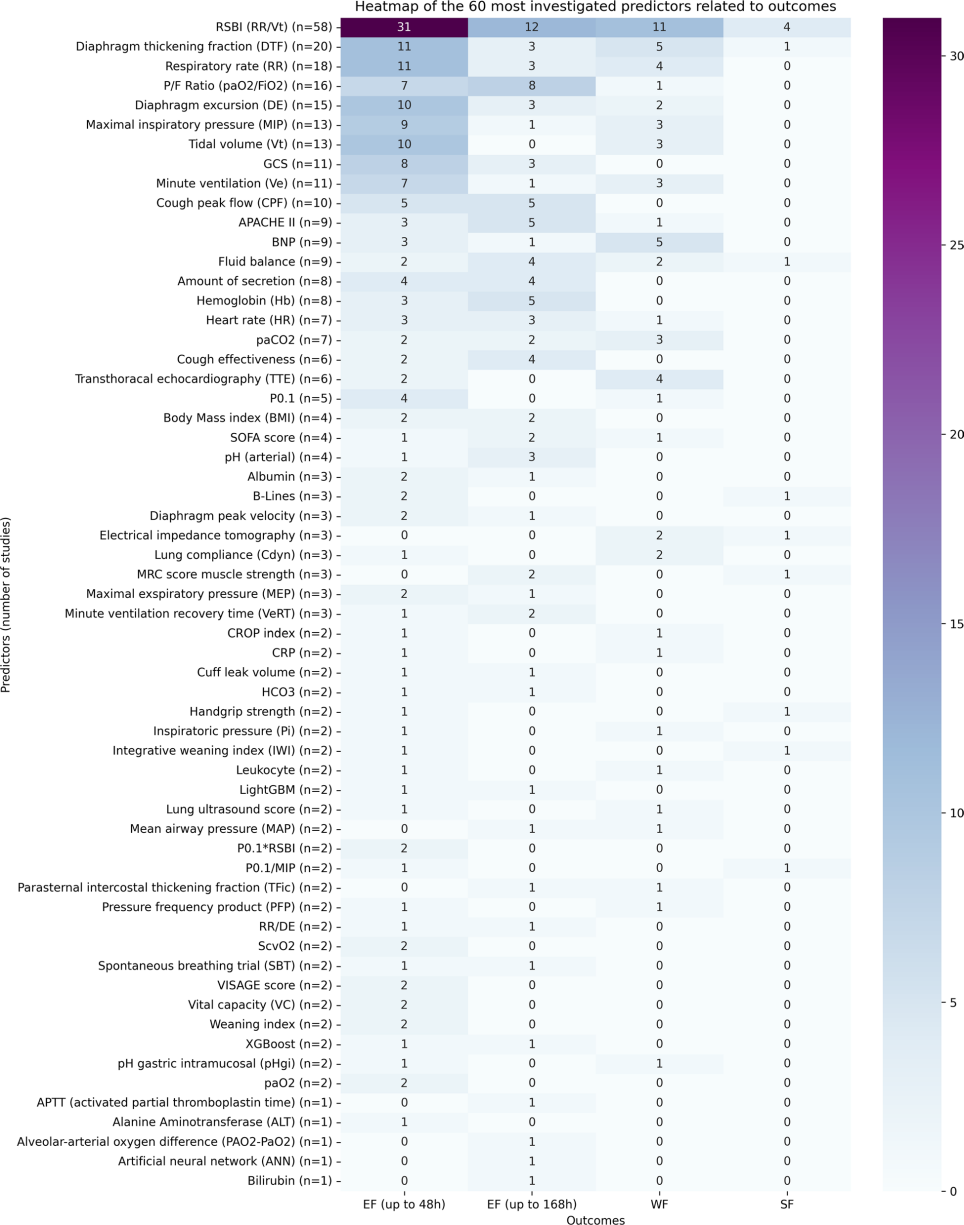
Heatmap of the 60 most investigated predictors related to outcomes

Further results can be derived from the description of the individual predictors. The heatmap illustrates which predictors were examined the most and further differentiates the number of studies along the respective outcomes. The RSBI, the DTF, the RR, the P/F ratio, and the DE are the most frequently investigated predictors, EF up to 48 h was the most frequently investigated outcome (see Fig. 8).

**Fig. 9:**
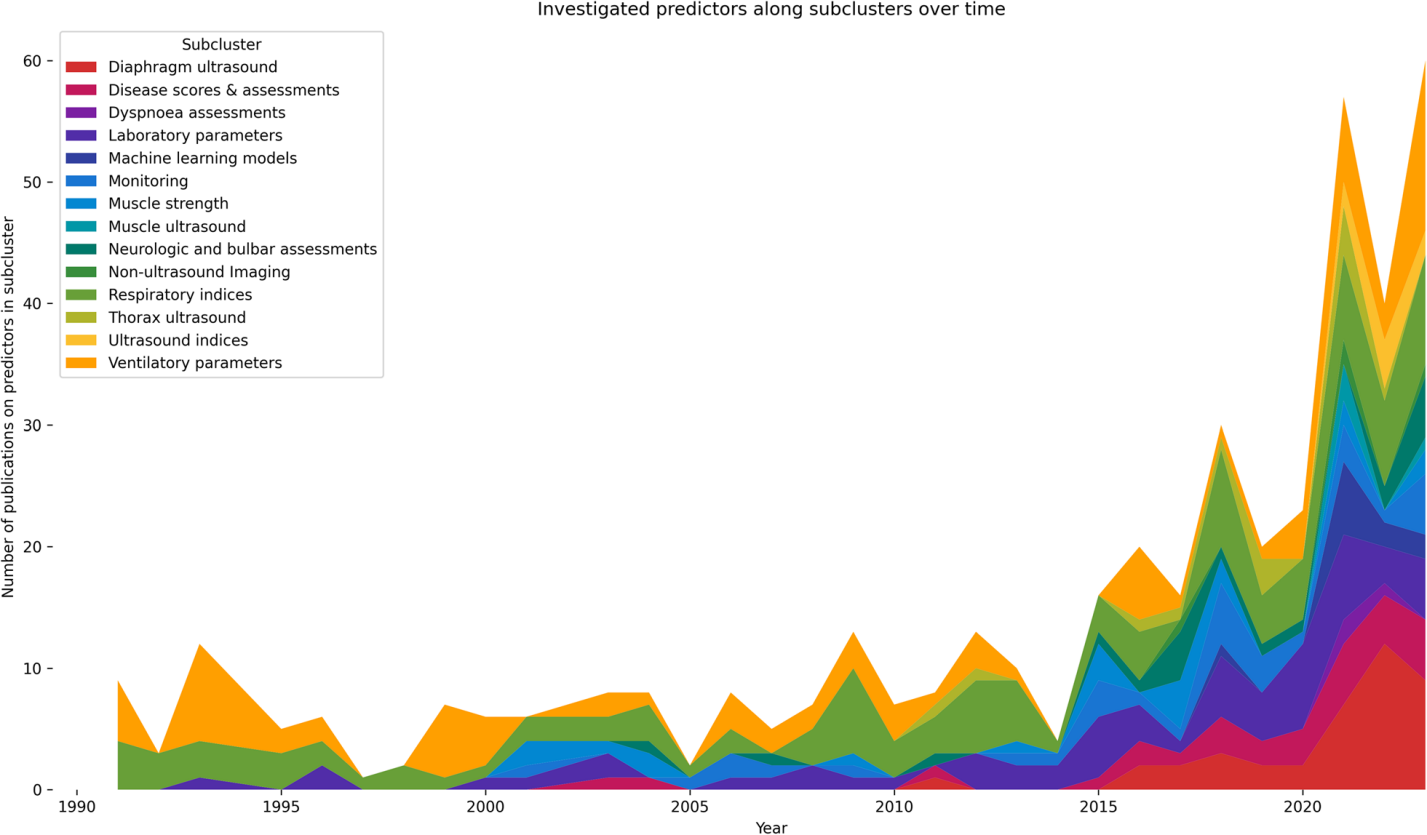
Investigated predictors along subclusters over time

The subcluster analysis highlights that the focus of conducted research has changed over the years. Whereas in the 1990s only ventilatory parameters, laboratory parameters, and respiratory indices were investigated as potential predictors, research interest in ultrasound examinations or monitoring has increased significantly in recent years. Also, the use of machine learning models has been examined increasingly in the last few years (see Fig. 9).

## Discussion

### Summary of evidence

The aim of this review was to provide a systematic overview of empirically investigated predictors for WF whose outcomes are in line with the current weaning definition [6]. To this end, we designed an evidence map and conducted systematic and complementary searches. A total of 140 studies were included, in which 145 predictors were identified and assigned to the four main clusters ‘Imaging procedures’ (n = 22), ‘Physiologic parameters’ (n = 61), ‘Scores and indices’ (n = 53), and ‘Machine learning models’ (n = 9).

The results of this review highlight the overall broad corpus of evidence in this research area. However, this extent was not always provided. Over the past decade, new parameters were being increasingly investigated as predictors and most of the included studies were published during this period (see Fig. 9). Nevertheless, the extent of the topic and the 145 predictors identified also highlight a complex evidence base, which reaffirms the importance of this review. Furthermore, it is not only the extent of the individual clusters that varies, but also how often individual predictors were examined. While the RSBI has already been tested for its predictive function in 58 studies, other factors were examined considerably less (see Fig. 8). In detail, 85 factors (e.g., ALT, ANN, APTT) have only been tested once and 24 factors have only been tested twice so far. Thus, there is a clear lack of updates for the majority of the identified predictors in this review.

A number of studies are now also available on machine learning models. These differ significantly in their complexity from other studies, in which the predictors are usually based on one or a few parameters. Machine learning models rely on a much larger amount of data, incorporating e.g. 28 factors [150], 37 factors [82] or 57 factors [170]. This may explain, why these models often result in an area under the curve of 0.85 or higher [82, 100, 150, 168, 170] and therefore perform better than other predictors (see Additional File 3).

As our review asked for predictors in general, no restrictions were made regarding the patient cohort. This resulted in a heterogeneous population in the studies. For example, studies included COVID-19 patients [55, 76, 92], neurocritical patients [91, 138], or surgical patients [107]. Accordingly, our study cannot draw conclusions for specific populations. However, several systematic reviews and meta-analyses have been published that deal with individual predictors such as BNP [17, 171] and diaphragm ultrasound [172–174], or specific populations such as neurocritical patients [18, 175].

As shown in the results, the included studies examined the predictors concerning various outcomes. Although the outcomes SBT failure and EF can be clearly separated from each other, they are defined heterogeneously in the included studies. For instance, EF was defined as reintubation within 24 h (e.g., [80]), 48 h (e.g., [85]), 72 h (e.g., [33]), 5 days (e.g., [167]) or 7 days (e.g., [118]). In addition, some studies also defined patient death as EF (e.g., [51, 82]) while others did not. Furthermore, SBT failure and EF are merged under the label WF in 29 studies and cause heterogeneity in the comparison group. These approaches raise the question of whether the investigated predictors can be applied to the individuals in their specific treatment situation. Moreover, no studies could be included that examined the outcome DF according to our definition. In all identified studies on DF, some or all of the patients were already weaned from their ventilatory support.

There are also methodological differences in the interpretation of the results. Despite the question of whether a factor is actually predictive was not the subject of this review, the data extraction revealed that this question cannot be answered generally with yes or no, but rather requires an interpretation of 'more or less predictive'. However, we found considerable heterogeneity in the judgement of the predictive function of individual factors in the studies, as also found in other studies [176]. Therefore, we refrained from interpreting the results and only presented the raw data in our study characteristics table (see Additional file 3).

### Limitations

Our review also has several limitations. First, we only included German and English studies, hence articles in other languages were excluded from our review. Second, we cannot rule out a publication bias. Third, the methodological distinction between predictive and explanatory factors were not always clear-cut, as many studies do not differentiate between them as we do [27]. Nevertheless, we believe that this distinction is of central importance. Accordingly, our common understanding enabled us to resolve any ambiguities together. Fourth, we did not appraise the study quality. Although this is in line with the scoping review method, it does not allow any statement about the accuracy of the individual studies and the predictors investigated therein. Fifth, a large number of other studies were excluded from our evidence map because they defined NIV as WF and were therefore not in line with our inclusion criteria. Compared to the previous ICC definition of weaning failure [19], the current definition of the WIND study no longer considers NIV as a WF [6]. Based on this criterion alone, we excluded 93 studies from our review (see Additional file 2). It is also remarkable that 63 of these 93 studies were published in 2018 or later (e.g., (177, 178). It remains unclear why the more recent studies do not take the current definition into account. As a consequence, our review can only serve as a literature review in the light of the current weaning definition.

## Conclusion

The overall field of predictors for WF in ICU patients undergoing MV is widely researched. In this review, 140 studies reveal 145 predictors, which have been investigated with varying intensity. In recent years in particular, new predictors have been investigated (e.g., imaging procedures). Machine learning models that combine a variety of factors seem particularly promising.

For clinicians caring for weaning patients, factors to predict weaning failure remain of great importance. However, although there is a large number of predictors, only a few of them appear to be robust and reliable. To ensure patient safety, clinicians should therefore rely on the few that are supported by a broad evidence base. In addition, clinicians should consider not only one, but several predictors in their assessment and evaluation of weaning patients.

Future research has various tasks. As a large number of predictors have only been tested in pilot studies, their predictive function needs to be confirmed in larger prospective studies (see Fig. 8). In addition, meta-analyses should be carried out to compare the quality of available studies and the reported effectiveness of predictors within studies at a higher level and to derive further insights. Finally, research should also follow the existing and internationally consented definitions. Although this limits comparability with older studies, research will no longer be conducted based on outdated eligibility criteria and be comparable with current studies.

## Supplementary Information

Additional file1 (PDF 196 KB) This supplement contains the entire search strategies and the results of individual searches and sources

Additional file2 (PDF 383 KB) This supplement provides details on 154 studies, which were excluded during full-text screening

Additional file3 (PDF 392 KB) This supplement contains the study characteristics table and further information extracted from the 140 included original studies

## Data Availability

The datasets supporting the conclusions of this article are included within the article and its additional files. Further data used and/or analyzed during the current study are available from the corresponding author.

## Abbreviations

AChE: Red blood cell acetylcholinesterase
ALT: Alanine aminotransferase
ANN: Artificial neural network
BNP: B-type natriuretic peptide
BUN: Blood urea nitrogen level
CCI: Charlson comorbidity index
CFS: Clinical frailty score
CINAHL: Cumulative index to nursing and allied health literature
CNN: Convolutional neural network
CPF: Cough peak flow
CVP: Central venous pressure
DE: Diaphragm excursion
DF: Decannulation failure
DLS: Diaphragm longitudinal strain
DTF: Diaphragm thickening fraction
EF: Extubation failure
FRC: Functional residual capacity
GCS: Glasgow coma scale
Hb: Hemoglobin
HR: Heart rate
HRV: Heart rate variability
ICC: International consensus conference of intensive care medicine
ICU: Intensive care unit
IEQ: Inspiratory effort quotient
IV-RDOS: Intensive care respiratory distress observational scale
IWI: Integrative weaning index
LIS: Lung injury score
MAP: Mean airway pressure
MBP: Mean blood pressure
MDA: Malondialdehyde
MEP: Maximal expiratory pressure
MIP: Maximal inspiratory pressure
MP: Mechanical power
MPV: Mean platelet volume
MV: Mechanical ventilation
MV-RDOS: Mechanical ventilation-respiratory distress observation scale
NIV: Non-invasive ventilation
ΔP: Driving pressure
pBW: Predicted body weight
PCED: Passive cephalic excursion of the diaphragm
PEEP: Positive end-expiratory pressure
PFP: Pressure frequency product
Pi: Inspiratory pressure
PRISMA: Preferred reporting items for systematic reviews and meta-analyses
PRISMA-ScR: PRISMA extension for scoping reviews
Ptr,stim: Twitch tracheal pressure in response to magnetic phrenic stimulation
RISC: Reintubation scale calculation score
RIS-I: Respiratory insufficiency scale-intubated
RR: Respiratory rate
RSBI: Rapid shallow breathing index
RSDI: Rapid shallow diaphragmatic index
SBT: Spontaneous breathing trial
SChE: Serum cholinesterase
SCSS: Semi-quantitative cough strength score
SEM: Systematic evidence map
TBSA: Total body surface are burned
TEE: Transesophageal echocardiography
TFC: Thoracic fluid content
TFic: Parasternal intercostal thickening fraction
TIE: Timed inspiratory effort index
TISS: Therapeutic intervention scoring system
Trf: Thickness of musculus rectus femoris
TTE: Transthoracic echocardiography
TTI: Tension time index
Tvi: Thickness of musculus vastus intermedius
VC: Vital capacity
Ve: Minute ventilation
VeRT: Minute ventilation recovery time
Vt: Tidal volume
WOB: Work of breathing
