# Supplementary material for: Predictors of weaning failure in ventilated intensive care patients: a systematic evidence map
Source: Crit Care. 2024 Nov 12;28:366. doi: 10.1186/s13054-024-05135-3 (PMC11556093; doi:10.1186/s13054-024-05135-3)
Supplement: Supplementary file 1 — Additional file1 (PDF 196 KB) This supplement contains the entire search strategies and the results of individual searches and sources [file 13054_2024_5135_MOESM1_ESM.pdf]

Additional file 1: Search process

Review on predictors of ventilator weaning failure

Systematic searches within databases

Medline (via PubMed)<br>Date of search: 12/19/2023			
**Content**	**Number**	**Keyword(s)**	**Results**
**Weaning failure**	#1	"weaning fail*"[Title/Abstract]	601
	#2	"extubation fail*"[Title/Abstract]	929
	#3	"decannulation fail*"[Title/Abstract]	57
	**#4**	**#1-#3/OR**<br>(("weaning fail*"[Title/Abstract]) OR ("extubation fail*"[Title/Abstract])) OR ("decannulation fail*"[Title/Abstract])	1,542
**Predictor**	#5	predict*[Title/Abstract]	2,115,730
	#6	risk factors[MeSH Terms]	971,260
	#7	risk factor*[Title/Abstract]	794,440
	#8	independent variabl*[Title/Abstract]	36,191
	#9	logistic regression[Title/Abstract]	413,741
	#10	logistic model[MeSH Terms]	152,023
	**#11**	**#5-#10/OR**<br>(((((predict*[Title/Abstract]) OR (risk factors[MeSH Terms])) OR (risk factor*[Title/Abstract])) OR (independent variabl*[Title/Abstract])) OR (logistic regression[Title/Abstract])) OR (logistic model[MeSH Terms])	3,481,461
**Artificial respiration**	#12	artificial respiration[MeSH Terms]	89,817
	#13	mechanical* ventilat*[Title/Abstract]	79,411
	#14	invasive* ventilat*[Title/Abstract]	19,198
	#15	artificial* ventilat*[Title/Abstract]	47,404
	#16	invasive* respir*[Title/Abstract]	20,993
	**#17**	**#12-#16/OR**<br>((((artificial respiration[MeSH Terms]) OR (mechanical* ventilat*[Title/Abstract])) OR (invasive* ventilat*[Title/Abstract])) OR (artificial* ventilat*[Title/Abstract])) OR (invasive* respir*[Title/Abstract])	158,065
**Combined**	**#18**	**#4 AND #11 AND #17**<br>(((("weaning fail*"[Title/Abstract]) OR ("extubation fail*"[Title/Abstract])) OR ("decannulation fail*"[Title/Abstract])) AND ((((((predict*[Title/Abstract]) OR (risk factors[MeSH Terms])) OR (risk factor*[Title/Abstract])) OR (independent variabl*[Title/Abstract])) OR (logistic regression[Title/Abstract])) OR (logistic model[MeSH Terms]))) AND (((((artificial respiration[MeSH Terms]) OR (mechanical* ventilat*[Title/Abstract])) OR (invasive* ventilat*[Title/Abstract])) OR (artificial* ventilat*[Title/Abstract])) OR (invasive* respir*[Title/Abstract]))	677

Cochrane Library<br>Date of search: 12/19/2023			
**Content**	**Number**	**Keyword(s)**	**Results**
**Weaning failure**	#1	weaning fail*[Title/Abstract/Keyword]	1,160
	#2	extubation fail*[Title/Abstract/Keyword]	1,368
	#3	decannulation fail*[Title/Abstract/Keyword]	24
	**#4**	**#1-#3/OR**<br>(weaning fail*[Title/Abstract/Keyword]) OR (extubation fail*[Title/Abstract/Keyword]) OR (decannulation fail*[Title/Abstract/Keyword])	2,107
**Predictor**	#5	predict*[Title/Abstract/Keyword]	119,302
	#6	risk factors[MeSH descriptor]	33,304
	#7	risk factor*[Title/Abstract/Keyword]	119,504
	#8	independent variabl*[Title/Abstract/Keyword]	10,777
	#9	logistic regression[Title/Abstract/Keyword]	25,960
	#10	logistic models[MeSH descriptor]	6,399
	**#11**	**#5-#10/OR**<br>(predict*[Title/Abstract/Keyword]) OR (risk factors[MeSH descriptor]) OR (risk factor*[Title/Abstract/Keyword]) OR (independent variabl*[Title/Abstract/Keyword]) OR (logistic regression[Title/Abstract/Keyword]) OR (logistic models[MeSH descriptor])	238,490
**Artificial respiration**	#12	Respiration, Artificial[MeSH descriptor]	8,350
	#13	mechanical* ventilat*[Title/Abstract/Keyword]	16,289
	#14	invasive* ventilat*[Title/Abstract/Keyword]	5,839
	#15	artificial* ventilat*[Title/Abstract/Keyword]	11,317
	#16	invasive* respir*[Title/Abstract/Keyword]	5,438
	**#17**	**#12-#16/OR**<br>((((artificial respiration[MeSH descriptor]) OR (mechanical* ventilat*[Title/Abstract/Keyword])) OR (invasive* ventilat*[Title/Abstract/Keyword])) OR (artificial* ventilat*[Title/Abstract/Keyword])) OR (invasive* respir*[Title/Abstract/Keyword])	28,013
**Combined**	**#18**	**#4 AND #11 AND #17**<br>((weaning fail*[Title/Abstract/Keyword]) OR (extubation fail*[Title/Abstract/Keyword]) OR (decannulation fail*[Title/Abstract/Keyword])) AND ((predict*[Title/Abstract/Keyword]) OR (risk factors[MeSH descriptor]) OR (risk factor*[Title/Abstract/Keyword]) OR (independent variabl*[Title/Abstract/Keyword]) OR (logistic regression[Title/Abstract/Keyword]) OR (logistic models[MeSH descriptor])) AND (((((artificial respiration[MeSH descriptor]) OR (mechanical* ventilat*[Title/Abstract/Keyword])) OR (invasive* ventilat*[Title/Abstract/Keyword])) OR (artificial* ventilat*[Title/Abstract/Keyword])) OR (invasive* respir*[Title/Abstract/Keyword]))	324

CINAHL (via EBSCOhost)<br>Date of search: 12/19/2023			
**Content**	**Number**	**Keyword(s)**	**Results**
**Weaning failure**	#1	weaning fail*	452
	#2	extubation fail*	685
	#3	decannulation fail*	41
	**#4**	**#1-#3/OR**<br>weaning fail* OR extubation fail* OR decannulation fail*	1,111
**Predictor**	#5	predict*	134,339
	#6	(MH "Risk Factors")	199,591
	#7	risk factor*	586,370
	#8	(MH "Independent Variable")	4,023
	#9	(MH "Logistic Regression")	141,798
	**#10**	**#5-#9/OR**<br>predict* OR (MH "Risk Factors") OR risk factor* OR (MH "Independent Variable") OR (MH "Logistic Regression")	1,060,622
**Artificial respiration**	#11	(MH "Respiration, Artificial")	25,807
	#12	mechanical* ventilat*	26,876
	#13	invasive* ventilat*	4,335
	#14	artificial* ventilat*	614
	#15	invasive* respir*	920
	**#16**	**#11-#15/OR**<br>(MH "Respiration, Artificial") OR mechanical* ventilat* OR invasive* ventilat* OR artificial* ventilat* OR invasive* respir*	42,365
**Combined**	**#17**	**#4 AND #10 AND #16**<br>(weaning fail* OR extubation fail* OR decannulation fail*) AND (predict* OR (MH "Risk Factors") OR risk factor* OR (MH "Independent Variable") OR (MH "Logistic Regression")) AND ((MH "Respiration, Artificial") OR mechanical* ventilat* OR invasive* ventilat* OR artificial* ventilat* OR invasive* respir*)	356

02. Additional searches

Source	Search string	Filters	Relevant Results	Date of search
LIVIVO	predictor weaning failure	None	4	06/10/2024
LIVIVO	predicition weaning failure	None		06/10/2024
LIVIVO	predictor extubation failure	None		06/10/2024
LIVIVO	prediction extubation failure	None		06/10/2024
LIVIVO	predictor decannulation failure	None		06/10/2024
LIVIVO	prediction decannulation failure	None		06/10/2024
Google Scholar	predictor weaning failure	None	2	07/10/2024
Google Scholar	predicition weaning failure	None		07/10/2024
Google Scholar	predictor extubation failure	None		07/10/2024
Google Scholar	prediction extubation failure	None		07/10/2024
Google Scholar	predictor decannulation failure	None		07/10/2024
Google Scholar	prediction decannulation failure	None		07/10/2024

03. Citation search

Relevant results obtained: **25**

Seed references for backward and forward citation searching:

- Cao, J., Wang, B., Zhu, L., & Song, L. (2022). Pooled Analysis of Central Venous Pressure and Brain Natriuretic Peptide Levels in Patients With Extubation Failure. Frontiers in physiology, 13, 858046. https://doi.org/10.3389/fphys.2022.858046
- da Silva, A. R., Novais, M. C. M., Neto, M. G., & Correia, H. F. (2023). Predictors of extubation failure in neurocritical patients: A systematic review. Australian critical care : official journal of the Confederation of Australian Critical Care Nurses, 36(2), 285–291. https://doi.org/10.1016/j.aucc.2021.11.005
- Jia, D., Wang, H., Wang, Q., Li, W., Lan, X., Zhou, H., & Zhang, Z. (2024). Rapid shallow breathing index predicting extubation outcomes: A systematic review and meta-analysis. Intensive & critical care nursing, 80, 103551. https://doi.org/10.1016/j.iccn.2023.103551
- Le Neindre, A., Philippart, F., Luperto, M., Wormser, J., Morel-Sapene, J., Aho, S. L., Mongodi, S., Mojoli, F., & Bouhemad, B. (2021). Diagnostic accuracy of diaphragm ultrasound to predict weaning outcome: A systematic review and meta-analysis. International journal of nursing studies, 117, 103890. https://doi.org/10.1016/j.ijnurstu.2021.103890
- Li, C., Li, X., Han, H., Cui, H., Wang, G., & Wang, Z. (2018). Diaphragmatic ultrasonography for predicting ventilator weaning: A meta-analysis. Medicine, 97(22), e10968. https://doi.org/10.1097/MD.0000000000010968
- Liu, J., Wang, C. J., Ran, J. H., Lin, S. H., Deng, D., Ma, Y., & Xu, F. (2021). The predictive value of brain natriuretic peptide or N-terminal pro-brain natriuretic peptide for weaning outcome in mechanical ventilation patients: Evidence from SROC. Journal of the renin-angiotensin-aldosterone system : JRAAS, 22(1), 1470320321999497. https://doi.org/10.1177/1470320321999497

- Lombardi, F. S., Cotoia, A., Petta, R., Schultz, M., Cinnella, G., & Horn, J. (2019). Prediction of extubation failure in Intensive Care Unit: systematic review of parameters investigated. Minerva anestesiologica, 85(3), 298–307. https://doi.org/10.23736/S0375-9393.18.12627-7
- Mahmoodpoor, A., Fouladi, S., Ramouz, A., Shadvar, K., Ostadi, Z., & Soleimanpour, H. (2022). Diaphragm ultrasound to predict weaning outcome: systematic review and meta-analysis. Anaesthesiology intensive therapy, 54(2), 164–174. https://doi.org/10.5114/ait.2022.117273
- de Meirelles Almeida, C. A., Nedel, W. L., Morais, V. D., Boniatti, M. M., & de Almeida-Filho, O. C. (2016). Diastolic dysfunction as a predictor of weaning failure: A systematic review and meta-analysis. Journal of critical care, 34, 135–141. https://doi.org/10.1016/j.jcrc.2016.03.007
- Nemer, S. N., & Barbas, C. S. (2011). Predictive parameters for weaning from mechanical ventilation. Jornal brasileiro de pneumologia : publicacao oficial da Sociedade Brasileira de Pneumologia e Tisilogia, 37(5), 669–679. https://doi.org/10.1590/s1806-37132011000500016
- Qian, Z., Yang, M., Li, L., & Chen, Y. (2018). Ultrasound assessment of diaphragmatic dysfunction as a predictor of weaning outcome from mechanical ventilation: a systematic review and meta-analysis. BMJ open, 8(9), e021189. https://doi.org/10.1136/bmjopen-2017-021189
- Torrini, F., Gendreau, S., Morel, J., Carteaux, G., Thille, A. W., Antonelli, M., & Mekontso Dessap, A. (2021). Prediction of extubation outcome in critically ill patients: a systematic review and meta-analysis. Critical care (London, England), 25(1), 391. https://doi.org/10.1186/s13054-021-03802-3
- Wang, S., Zhang, L., Huang, K., Lin, Z., Qiao, W., & Pan, S. (2014). Predictors of extubation failure in neurocritical patients identified by a systematic review and meta-analysis. PloS one, 9(12), e112198. https://doi.org/10.1371/journal.pone.0112198
- Wu, C., Hu, L., Shen, Q., Xu, H., & Huang, H. (2023). Predictive value of extubation failure by decrease in central venous oxygen saturation: A systematic review and meta-analysis. Heliyon, 9(7), e18227. https://doi.org/10.1016/j.heliyon.2023.e18227
- Zambon, M., Greco, M., Bocchino, S., Cabrini, L., Beccaria, P. F., & Zangrillo, A. (2017). Assessment of diaphragmatic dysfunction in the critically ill patient with ultrasound: a systematic review. Intensive care medicine, 43(1), 29–38. https://doi.org/10.1007/s00134-016-4524-z
