# Supplementary material for: Predictors of weaning failure in ventilated intensive care patients: a systematic evidence map
Source: Crit Care. 2024 Nov 12;28:366. doi: 10.1186/s13054-024-05135-3 (PMC11556093; doi:10.1186/s13054-024-05135-3)
Supplement: Supplementary file 2 — Additional file2 (PDF 383 KB) This supplement provides details on 154 studies, which were excluded during full-text screening [file 13054_2024_5135_MOESM2_ESM.pdf]

Additional file 2: Excluded studies in fulltext screening

Systematic evidence map on predictors of weaning failure

Nr.	Excluded study	Reason
1	Abbas, A., Embarak, S., Walaa, M., & Lutfy, S. M. (2018). Role of diaphragmatic rapid shallow breathing index in predicting weaning outcome in patients with acute exacerbation of COPD. International journal of chronic obstructive pulmonary disease, 13, 1655–1661. https://doi.org/10.2147/COPD.S161691	Wrong outcome: NIV as extubation failure
2	Al Tayar, A. S., & Abdelshafey, E. E. (2022). Diaphragm Electromyography Versus Ultrasonography in the Prediction of Mechanical Ventilation Liberation Outcome. Respiratory care, respcare.09779. Advance online publication. https://doi.org/10.4187/respcare.09779	Wrong outcome: NIV as extubation failure
3	Alansary, A. M., & Hakim, K. (2020). Role of lung ultrasound in weaning from mechanical ventilation in postoperative neurosurgical ICU patients. *Sri Lankan Journal of Anaesthesiology, 28*(1), 25-30. http://dx.doi.org/10.4038/slja.v28i1.8481	Wrong outcome: NIV as extubation failure
4	Aldabayan, Y. S., Tolba, A. A., Alrajeh, A. M., Ahmed, A. T., Mahgoub, A. A., Glalah, A. A. A., & Abdelhafez, A. I. (2023). Factors Affecting Mechanical Ventilator Weaning Success and 28-Day Survival Among Patients With Acute Respiratory Distress Syndrome Secondary to COVID-19. SAGE open nursing, 9, 23779608231187248. https://doi.org/10.1177/23779608231187248	Wrong outcome: Article focuses on success only; there is no clear distinction between success and failure (e.g. is an RR of 35 in the SBT a failure or success? Allocation missing)
5	Ali, E. R., & Mohamad, A. M. (2017). Diaphragm ultrasound as a new functional and morphological index of outcome, prognosis and discontinuation from mechanical ventilation in critically ill patients and evaluating the possible protective indices against VIDD. Egyptian Journal of Chest Diseases and Tuberculosis, 66(2), 339-351. https://doi.org/10.1016/j.ejcdt.2016.10.006	Wrong outcome: NIV as extubation failure
6	Alvisi, R., Volta, C. A., Righini, E. R., Capuzzo, M., Ragazzi, R., Verri, M., Candini, G., Gritti, G., & Milic-Emili, J. (2000). Predictors of weaning outcome in chronic obstructive pulmonary disease patients. The European respiratory journal, 15(4), 656–662. https://doi.org/10.1034/j.1399-3003.2000.15d06.x	Wrong scope: Investigators' aim (investigation of threshold values) does not fit our aim, no focus on identification of predictors
7	Anderson, C. D., Bartscher, J. F., Scripko, P. D., Biffi, A., Chase, D., Guanci, M., & Greer, D. M. (2011). Neurologic examination and extubation outcome in the neurocritical care unit. Neurocritical care, 15(3), 490–497. https://doi.org/10.1007/s12028-010-9369-7	Wrong study design: No clear distinction between explanatory and predictive factors

8	Baess, A. I., Abdallah, T. H., Emara, D. M., & Hassan, M. (2016). Diaphragmatic ultrasound as a predictor of successful extubation from mechanical ventilation: thickness, displacement, or both? Egyptian Journal of Bronchology, 10, 12-166. https://doi.org/10.4103/1687-8426.184370	Wrong outcome: NIV as extubation failure
9	Baptistella, A. R., Mantelli, L. M., Matte, L., Carvalho, M. E. D. R. U., Fortunatti, J. A., Costa, I. Z., Haro, F. G., Turkot, V. L. O., Baptistella, S. F., de Carvalho, D., & Nunes Filho, J. R. (2021). Prediction of extubation outcome in mechanically ventilated patients: Development and validation of the Extubation Predictive Score (ExPreS). PloS one, 16(3), e0248868. https://doi.org/10.1371/journal.pone.0248868	Wrong outcome: NIV as extubation failure
10	Barwing, J., Pedroni, C., Olgemöller, U., Quintel, M., & Moerer, O. (2013). Electrical activity of the diaphragm (EAdi) as a monitoring parameter in difficult weaning from respirator: a pilot study. Critical care (London, England), 17(4), R182. https://doi.org/10.1186/cc12865	Wrong study design: Only group comparisons, no statistical calculations of probabilities
11	Baysal, O. F., Coskun, R., Büyükoglan, H., Sungur, M., & Güven, M. (2010). Can gastric tonometry be used to determine weaning failure? *Turkish Journal of Medical & Surgical Intensive Care Medicine*, 3, 55-59. https://doi.org/10.5152/dcbybd.2010.07	Wrong outcome: NIV as extubation failure
12	Beigmohammadi, M. T., Hussain Khan, Z., Samadi, S., Mahmoodpoor, A., Fotouhi, A., Rahimiforoushani, A., & Asadi Gharabaghi, M. (2016). Role of Hematocrit Concentration on Successful Extubation in Critically Ill Patients in the Intensive Care Units. Anesthesiology and pain medicine, 6(1), e32904. https://doi.org/10.5812/aapm.32904	Wrong study design: Only explanatory factors investigated in multiple logistic regression
13	Bien, M. Y., Hseu, S. S., Yien, H. W., Kuo, B. I., Lin, Y. T., Wang, J. H., & Kou, Y. R. (2004). Breathing pattern variability: a weaning predictor in postoperative patients recovering from systemic inflammatory response syndrome. Intensive care medicine, 30(2), 241–247. https://doi.org/10.1007/s00134-003-2073-8	Wrong outcome: NIV as extubation failure
14	Bien, M. Y., Shui Lin, Y., Shih, C. H., Yang, Y. L., Lin, H. W., Bai, K. J., Wang, J. H., & Ru Kou, Y. (2011). Comparisons of predictive performance of breathing pattern variability measured during T-piece, automatic tube compensation, and pressure support ventilation for weaning intensive care unit patients from mechanical ventilation. Critical care medicine, 39(10), 2253–2262. https://doi.org/10.1097/CCM.0b013e31822279ed	Wrong outcome: NIV as extubation failure
15	Bonny, V., Joffre, J., Gabarre, P., Urbina, T., Missri, L., Ladoire, M., Gasperment, M., Baudel, J. L., Guidet, B., Dumas, G., Maury, E., Brochard, L., & Ait-Oufella, H. (2023). Sonometric assessment of cough predicts extubation failure: SonoWean-a proof-of-concept study. Critical care (London, England), 27(1), 368. https://doi.org/10.1186/s13054-023-04653-w	Wrong outcome: NIV as extubation failure
16	Bouhemad, B., Mojoli, F., Nowobilski, N., Hussain, A., Rouquette, I., Guinot, P. G., & Mongodi, S. (2020). Use of combined cardiac and lung ultrasound to predict weaning failure in elderly, high-	Wrong outcome: NIV as extubation failure

	risk cardiac patients: a pilot study. Intensive care medicine, 46(3), 475–484. https://doi.org/10.1007/s00134-019-05902-9	
17	Brown, C. V., Daigle, J. B., Foulkrod, K. H., Brouillette, B., Clark, A., Czysz, C., Martinez, M., & Cooper, H. (2011). Risk factors associated with early reintubation in trauma patients: a prospective observational study. The Journal of trauma, 71(1), 37–42. https://doi.org/10.1097/TA.0b013e31821e0c6e	Wrong study design: Only group comparisons, no statistical calculations of probabilities
18	Burns, S. M., Fisher, C., Tribble, S. E., Lewis, R., Merrel, P., Conaway, M. R., & Bleck, T. P. (2012). The relationship of 26 clinical factors to weaning outcome. American journal of critical care : an official publication, American Association of Critical-Care Nurses, 21(1), 52–59. https://doi.org/10.4037/ajcc2012425	Wrong outcome: NIV as extubation failure
19	Caille, V., Amiel, J. B., Charron, C., Belliard, G., Vieillard-Baron, A., & Vignon, P. (2010). Echocardiography: a help in the weaning process. Critical care (London, England), 14(3), R120. https://doi.org/10.1186/cc9076	Wrong study design: Only group comparisons, no statistical calculations of probabilities
20	Cammarota, G., Boniolo, E., Santangelo, E., De Vita, N., Verdina, F., Crudo, S., Sguazzotti, I., Perucca, R., Messina, A., Zanoni, M., Azzolina, D., Navalesi, P., Longhini, F., Vetrugno, L., Bignami, E., Della Corte, F., Tarquini, R., De Robertis, E., & Vaschetto, R. (2021). Diaphragmatic Kinetics Assessment by Tissue Doppler Imaging and Extubation Outcome. Respiratory care, 66(6), 983–993. https://doi.org/10.4187/respcare.08702	Wrong outcome: NIV as extubation failure
21	Cao, J., Wang, B., Zhu, L., & Song, L. (2022). Pooled Analysis of Central Venous Pressure and Brain Natriuretic Peptide Levels in Patients With Extubation Failure. Frontiers in physiology, 13, 858046. https://doi.org/10.3389/fphys.2022.858046	Wrong outcome: Studies included in meta-analysis refer to NIV as extubation failure
22	Carrera, M., Urrutia, J. G., Ardariz, C. B., Porra, M. L., Gamarra, C., & Ballve, L. P. D. (2023). Maximal expiratory pressure compared with maximal expiratory pressure during induced cough as a predictor of extubation failure. Critical care science, 35(1), 37–43. https://doi.org/10.5935/2965-2774.20230275-en	Wrong outcome: NIV as extubation failure
23	Carrie, C., Gisbert-Mora, C., Bonnardel, E., Gauche, B., Biais, M., Vargas, F., & Hilbert, G. (2017). Ultrasonographic diaphragmatic excursion is inaccurate and not better than the MRC score for predicting weaning-failure in mechanically ventilated patients. Anaesthesia, critical care & pain medicine, 36(1), 9–14. https://doi.org/10.1016/j.accpm.2016.05.009	Wrong outcome: NIV as extubation failure
24	Chung, W. C., Sheu, C. C., Hung, J. Y., Hsu, T. J., Yang, S. H., & Tsai, J. R. (2020). Novel mechanical ventilator weaning predictive model. The Kaohsiung journal of medical sciences, 36(10), 841–849. https://doi.org/10.1002/kjm2.12269	Wrong outcome: NIV as extubation failure

25	Cork, G., Camporota, L., Osman, L., & Shannon, H. (2019). Physiotherapist prediction of extubation outcome in the adult intensive care unit. Physiotherapy research international : the journal for researchers and clinicians in physical therapy, 24(4), e1793. https://doi.org/10.1002/pri.1793	Missing methodology: Insufficient description of methodological approach, wrong conclusions
26	Cottereau, G., Dres, M., Avenel, A., Fichet, J., Jacobs, F. M., Prat, D., Hamzaoui, O., Richard, C., Antonello, M., & Sztrymf, B. (2015). Handgrip Strength Predicts Difficult Weaning But Not Extubation Failure in Mechanically Ventilated Subjects. Respiratory care, 60(8), 1097–1104. https://doi.org/10.4187/respcare.03604	Wrong outcome: NIV as extubation failure
27	Cottereau, G., Messika, J., Megarbane, B., Guérin, L., da Silva, D., Bornstain, C., Santos, M., Ricard, J. D., & Sztrymf, B. (2021). Handgrip strength to predict extubation outcome: a prospective multicenter trial. Annals of intensive care, 11(1), 144. https://doi.org/10.1186/s13613-021-00932-3	Wrong outcome: NIV as extubation failure
28	da Silva, R. B., Neves, V. R., Montarroyos, U. R., Silveira, M. S., & Sobral Filho, D. C. (2023). Heart rate variability as a predictor of mechanical ventilation weaning outcomes. Heart & lung : the journal of critical care, 59, 33–36. https://doi.org/10.1016/j.hrtlng.2023.01.007	Wrong study design: Only group comparisons, no statistical calculations of probabilities
29	da Silva, A. R., Novais, M. C. M., Neto, M. G., & Correia, H. F. (2023). Predictors of extubation failure in neurocritical patients: A systematic review. Australian critical care : official journal of the Confederation of Australian Critical Care Nurses, 36(2), 285–291. https://doi.org/10.1016/j.aucc.2021.11.005	Wrong study design: Only systematic review, no new results
30	de Meirelles Almeida, C. A., Nedel, W. L., Morais, V. D., Boniatti, M. M., & de Almeida-Filho, O. C. (2016). Diastolic dysfunction as a predictor of weaning failure: A systematic review and meta-analysis. Journal of critical care, 34, 135–141. https://doi.org/10.1016/j.jcrc.2016.03.007	Wrong study design: Meta-analysis included only group comparisons, no calculation of probabilities
31	de Souza, L. C., Guimarães, F. S., & Lugon, J. R. (2015). The timed inspiratory effort: a promising index of mechanical ventilation weaning for patients with neurologic or neuromuscular diseases. Respiratory care, 60(2), 231–238. https://doi.org/10.4187/respcare.03393	Wrong outcome: Respiratory failure as an outcome until 48h of extubation, but no reintubation as recommended by our underlying definition (Bedunéau et al., 2017).
32	de Souza, L. C., Guimarães, F. S., & Lugon, J. R. (2015). Evaluation of a new index of mechanical ventilation weaning: the timed inspiratory effort. Journal of intensive care medicine, 30(1), 37–43. https://doi.org/10.1177/0885066613483265	Wrong outcome: Respiratory failure as an outcome until 48h of extubation, but no reintubation as recommended by our underlying definition (Bedunéau et al., 2017).
33	Deivert, M. M., Quatrara, B., Kane, C., Sohn, M. W., Childress, M. B., & Turrentine, F. E. (2019). An Evaluation of Risk Factors for Extubation Failure in Surgical Patients in Intensive Care.	Wrong outcome: Extubation failure included all reintubations within 30 days

	Dimensions of critical care nursing : DCCN, 38(5), 256–263. https://doi.org/10.1097/DCC.0000000000000380	
34	DiNino, E., Gartman, E. J., Sethi, J. M., & McCool, F. D. (2014). Diaphragm ultrasound as a predictor of successful extubation from mechanical ventilation. Thorax, 69(5), 423–427. https://doi.org/10.1136/thoraxjnl-2013-204111	Wrong outcome: Tracheostomy as extubation failure not in line with our definition
35	Domingues Angelo da Silva, L., Musumeci F. Almeida, M. M., Oliveira Quaresma, M., Castro, T., Ares Santos, M., & Dias Chiavegato, L. (2016). Success or failure predictive indexes of extubation in renal transplants patients under mechanical ventilation – pilot study. *Manual Therapy, Posturology & Rehabilitation Journal, 14*, 1-7. https://doi.org/10.17784/mtprehabjournal.2016.14.337	Wrong study design: Only group comparisons, no statistical calculations of probabilities
36	Dres, M., Schmidt, M., Ferre, A., Mayaux, J., Similowski, T., & Demoule, A. (2012). Diaphragm electromyographic activity as a predictor of weaning failure. Intensive care medicine, 38(12), 2017–2025. https://doi.org/10.1007/s00134-012-2700-3	Wrong outcome: NIV as extubation failure
37	Dubé, B. P., Dres, M., Mayaux, J., Demiri, S., Similowski, T., & Demoule, A. (2017). Ultrasound evaluation of diaphragm function in mechanically ventilated patients: comparison to phrenic stimulation and prognostic implications. Thorax, 72(9), 811–818. https://doi.org/10.1136/thoraxjnl-2016-209459	Wrong outcome: remaining time on MV and time to switch to PSV
38	El-Daim, A. A., El-Emery, F., El-Dib, A., & El-Shamaa, N. (2020). A study of different predictors of successful weaning off mechanical ventilation in ventilated patients with chronic obstructive pulmonary disease with acute respiratory failure. The Egyptian journal of chest diseases and tuberculosis, 69(3), 485-492. https://doi.org/10.4103/ejcdt.ejcdt_156_19	Missing methodology: Missing data on group characteristics, methodological approach and outcomes (definition of extubation failure)
39	Eltrabili, H. H., Hasanin, A. M., Soliman, M. S., Lotfy, A. M., Hamimy, W. I., & Mukhtar, A. M. (2019). Evaluation of Diaphragmatic Ultrasound Indices as Predictors of Successful Liberation From Mechanical Ventilation in Subjects With Abdominal Sepsis. Respiratory care, 64(5), 564–569. https://doi.org/10.4187/respcare.06391	Wrong outcome: NIV as extubation failure
40	Er, B., Mızrak, B., Aydemir, A., Binay, S., Doğu, C., Kazancı, D., & Turan, S. (2023). Is diaphragm ultrasound better than rapid shallow breathing index for predicting weaning in critically ill elderly patients?. Yoğun bakımdaki yaşlı kırılgan hastalarda diyafram ultrasonu mekanik ventilatörden ayrılmayı hızlı yüzeyel solunum indeksine göre daha iyi öngördürebilir mi?. Tuberkuloz ve toraks, 71(3), 197–202. https://doi.org/10.5578/tt.20239701	Wrong study design: Only group comparisons, no statistical calculations of probabilities
41	Farghaly, S., & Hasan, A. A. (2017). Diaphragm ultrasound as a new method to predict extubation outcome in mechanically ventilated patients. Australian critical care : official journal	Wrong outcome: NIV as extubation failure

	of the Confederation of Australian Critical Care Nurses, 30(1), 37–43. https://doi.org/10.1016/j.aucc.2016.03.004	
42	Fayed, A. M., Abd ElHady, M. A., Shaaban, M. S., & Fikry, D. M. (2016). Use of Ultrasound to Assess Diaphragmatic Thickness as a Weaning Parameter in Invasively Ventilated Chronic Obstructive Pulmonary Disease Patients. Journal of American Science, 12(6), 96-105. https://doi.org/10.7537/marsjas12061612	Wrong outcome: NIV as extubation failure
43	Ferrari, G., De Filippi, G., Elia, F., Panero, F., Volpicelli, G., & Aprà, F. (2014). Diaphragm ultrasound as a new index of discontinuation from mechanical ventilation. Critical ultrasound journal, 6(1), 8. https://doi.org/10.1186/2036-7902-6-8	Wrong setting: High dependency unit as a follow-up unit after ICU stay
44	Flevari, A., Lignos, M., Konstantonis, D., & Armaganidis, A. (2016). Diaphragmatic ultrasonography as an adjunct predictor tool of weaning success in patients with difficult and prolonged weaning. Minerva anestesiologica, 82(11), 1149–1157.	Wrong outcome: NIV as extubation failure
45	Ghamari, A. A., Amini, K., Daei Sorkhabi, A., Sarkesh, A., Saghaleini, S. H., Asghari, R., Rezayi, M., & Mahmoodpoor, A. (2023). Diagnostic value of an increase in central venous pressure during SBT for prediction of weaning failure in mechanically ventilated patients: A cross-sectional study. Health science reports, 6(4), e1204. https://doi.org/10.1002/hsr2.1204	Wrong study design: Only group comparisons, no statistical calculations of probabilities
46	Ghiani, A., Paderewska, J., Sainis, A., Crispin, A., Walcher, S., & Neurohr, C. (2020). Variables predicting weaning outcome in prolonged mechanically ventilated tracheotomized patients: a retrospective study. Journal of intensive care, 8, 19. https://doi.org/10.1186/s40560-020-00437-4	Wrong outcome: NIV as extubation failure
47	Ghiani, A., Paderewska, J., Walcher, S., & Neurohr, C. (2021). Mechanical power normalized to lung-thorax compliance predicts prolonged ventilation weaning failure: a prospective study. BMC pulmonary medicine, 21(1), 202. https://doi.org/10.1186/s12890-021-01566-8	Wrong outcome: NIV as extubation failure
48	Ghiani, A., Tsitouras, K., Paderewska, J., Milger, K., Walcher, S., Weiffenbach, M., Neurohr, C., & Kneidinger, N. (2022). Incidence, causes, and predictors of unsuccessful decannulation following prolonged weaning. Therapeutic advances in chronic disease, 13, 20406223221109655. https://doi.org/10.1177/20406223221109655	Wrong outcome: NIV as extubation failure
49	Godet, T., Chabanne, R., Marin, J., Kauffmann, S., Futier, E., Pereira, B., & Constantin, J. M. (2017). Extubation Failure in Brain-injured Patients: Risk Factors and Development of a Prediction Score in a Preliminary Prospective Cohort Study. Anesthesiology, 126(1), 104–114. https://doi.org/10.1097/ALN.0000000000001379	Wrong outcome: NIV as extubation failure

50	Gok, F., Mercan, A., Kilicaslan, A., Sarkilar, G., & Yosunkaya, A. (2021). Diaphragm and Lung Ultrasonography During Weaning From Mechanical Ventilation in Critically Ill Patients. Cureus, 13(5), e15057. https://doi.org/10.7759/cureus.15057	Wrong outcome: NIV as extubation failure
51	Gong, J., Zhang, B., Huang, X., Li, B., & Huang, J. (2021). Product of driving pressure and respiratory rate for predicting weaning outcomes. The Journal of international medical research, 49(5), 3000605211010045. https://doi.org/10.1177/03000605211010045	Wrong outcome: NIV as extubation failure
52	Grosu, H. B., Ost, D. E., Lee, Y. I., Song, J., Li, L., Eden, E., & Rose, K. (2017). Diaphragm Muscle Thinning in Subjects Receiving Mechanical Ventilation and Its Effect on Extubation. Respiratory care, 62(7), 904–911. https://doi.org/10.4187/respcare.05370	Wrong outcome: Tracheostomy as weaning failure is not in line with out inclusion criteria
53	Guerlain, J., Guerrero, J. A., Baujat, B., St Guily, J. L., & Périé, S. (2015). Peak inspiratory flow is a simple means of predicting decannulation success following head and neck cancer surgery: a prospective study of fifty-six patients. The Laryngoscope, 125(2), 365–370. https://doi.org/10.1002/lary.24904	Wrong setting: Not all patients were recruited in an ICU
54	Guzatti, N. G., Klein, F., Oliveira, J. A., Rático, G. B., Cordeiro, M. F., Marmitt, L. P., Carvalho, D., Nunes Filho, J. R., & Baptistella, A. R. (2022). Predictive Factors of Extubation Failure in COVID-19 Mechanically Ventilated Patients. Journal of intensive care medicine, 37(9), 1250–1255. https://doi.org/10.1177/08850666221093946	Wrong outcome: No clear cut-off for reintubation time (reintubation during ICU stay)
55	Haji, K., Haji, D., Canty, D. J., Royse, A. G., Green, C., & Royse, C. F. (2018). The impact of heart, lung and diaphragmatic ultrasound on prediction of failed extubation from mechanical ventilation in critically ill patients: a prospective observational pilot study. Critical ultrasound journal, 10(1), 13. https://doi.org/10.1186/s13089-018-0096-1	Wrong outcome: NIV as extubation failure
56	Hanneman, S. K. G. (1994). Multidimensional predictors of success or failure with early weaning from mechanical ventilation after cardiac surgery. *Nursing Research, 43*(1), 4-10. https://doi.org/10.1097/00006199-199401000-00002	Missing methodology: Unclear description of methodological approach during weaning (how and when was an SBT conducted and how was the procedure after failed/successful SBT?)
57	Hayat, A., Khan, A., Khalil, A., & Asghar, A. (2017). Diaphragmatic Excursion: Does it Predict Successful Weaning from Mechanical Ventilation? *Journal of the College of Physicians and Surgeons - Pakistan : JCPSP, 27*(12), 743–746. PMID: 29185398	Wrong outcome: NIV as extubation failure
58	He, G., Han, Y., Zhan, Y., Yao, Y., Zhou, H., & Zheng, X. (2023). The combined use of parasternal intercostal muscle thickening fraction and P0.1 for prediction of weaning outcomes. Heart & lung : the journal of critical care, 62, 122–128. https://doi.org/10.1016/j.hrtlng.2023.07.002	Wrong outcome: NIV as extubation failure

59	Hong, Y., Woo, S., Kim, Y., Lee, J. J., & Hong, J. Y. (2020). Plasma concentrations of NOX4 are predictive of successful liberation from mechanical ventilation and 28-day mortality in intubated patients. Annals of translational medicine, 8(21), 1376. https://doi.org/10.21037/atm-20-4252	Missing methodology: Inconsistent use of outcomes weaning failure, SBT failure, and extubation failure
60	Huang, C. T., & Yu, C. J. (2013). Conventional weaning parameters do not predict extubation outcome in intubated subjects requiring prolonged mechanical ventilation. Respiratory care, 58(8), 1307–1314. https://doi.org/10.4187/respcare.01773	Wrong setting: Intermediate respiratory care unit after ICU, patients with hemodynamic stability
61	Hurtado, F. J., Berón, M., Olivera, W., Garrido, R., Silva, J., Caragna, E., & Rivara, D. (2001). Gastric intramucosal pH and intraluminal PCO2 during weaning from mechanical ventilation. Critical care medicine, 29(1), 70–76. https://doi.org/10.1097/00003246-200101000-00017	Missing methodology: No definitions of central outcomes
62	Jabaudon, M., Perbet, S., Pereira, B., Soummer, A., Roszyk, L., Guérin, R., Futier, E., Lu, Q., Bazin, J. E., Sapin, V., Rouby, J. J., & Constantin, J. M. (2013). Plasma levels of sRAGE, loss of aeration and weaning failure in ICU patients: a prospective observational multicenter study. PloS one, 8(5), e64083. https://doi.org/10.1371/journal.pone.0064083	Wrong study design: Only group comparisons, no statistical calculations of probabilities
63	Jeong, B. H., Nam, J., Ko, M. G., Chung, C. R., Suh, G. Y., & Jeon, K. (2018). Impact of limb weakness on extubation failure after planned extubation in medical patients. Respirology (Carlton, Vic.), 10.1111/resp.13305 Titel anhand dieser DOI in Citavi-Projekt übernehmen. Advance online publication. https://doi.org/10.1111/resp.13305	Wrong outcome: NIV as extubation failure
64	Jia, D., Wang, H., Wang, Q., Li, W., Lan, X., Zhou, H., & Zhang, Z. (2024). Rapid shallow breathing index predicting extubation outcomes: A systematic review and meta-analysis. Intensive & critical care nursing, 80, 103551. https://doi.org/10.1016/j.iccn.2023.103551	Wrong outcome: NIV as extubation failure
65	Jiang, J., Tsai, T., Jerng, J., Yu, C., Wu, H., Yang, P., Jiang, J.-R., Tsai, T.-H., Jerng, J.-S., Yu, C.-J., Wu, H.-D., & Yang, P.-C. (2004). Ultrasonographic Evaluation of Liver/Spleen Movements and Extubation Outcome. *CHEST, 126*(1), 179-185. https://doi.org/10.1016/S0012-3692(15)32912-3	Wrong outcome: NIV as extubation failure
66	Jung, Y. T., Kim, M. J., Lee, J. G., & Lee, S. H. (2018). Predictors of early weaning failure from mechanical ventilation in critically ill patients after emergency gastrointestinal surgery: A retrospective study. Medicine, 97(40), e12741. https://doi.org/10.1097/MD.0000000000012741	Wrong outcome: Extubation rate only, no examination of reintubation or failed extubation
67	Karakurt, Z., Fanfulla, F., Ceriana, P., Carlucci, A., Grassi, M., Colombo, R., Karakurt, S., & Nava, S. (2012). Physiologic determinants of prolonged mechanical ventilation in patients after major surgery. Journal of critical care, 27(2), 221.e9–221.e2.21E16. https://doi.org/10.1016/j.jcrc.2011.08.009	Wrong setting: Weaning center with patients showing hemodynamic stability

68	Kaur, R., Alolaiwat, A. A., Ritz, E., Mokhlesi, B., & Vines, D. L. (2023). A new index, Respiratory Insufficiency index and Modified Early Warning Scores predict extubation failure. Canadian journal of respiratory therapy : CJRT = Revue canadienne de la therapie respiratoire : RCTR, 59, 117–122. https://doi.org/10.29390/cjrt-2023-003	Wrong outcome: NIV as extubation failure
69	Kaur, R., Vines, D. L., Liu, L., & Balk, R. A. (2017). Role of Integrated Pulmonary Index in Identifying Extubation Failure. Respiratory care, 62(12), 1550–1556. https://doi.org/10.4187/respcare.05434	Wrong outcome: NIV as extubation failure
70	Kaur, R., Vines, D. L., Patel, A. D., Lugo-Robles, R., & Balk, R. A. (2021). Early Identification of Extubation Failure Using Integrated Pulmonary Index and High-Risk Factors. Respiratory care, 66(10), 1542–1548. https://doi.org/10.4187/respcare.08656	Wrong outcome: NIV as extubation failure
71	Keim-Malpass, J., Enfield, K. B., Calland, J. F., Lake, D. E., & Clark, M. T. (2018). Dynamic data monitoring improves predictive analytics for failed extubation in the ICU. Physiological measurement, 39(7), 075005. https://doi.org/10.1088/1361-6579/aace95	Wrong outcome: Tracheostomy as weaning failure not in line with our definition
72	Khan, M. T., Munawar, K., Hussain, S. W., Qadeer, A., Saeed, M. L., Shad, Z. S., Qureshi, M. S. S., & Abdullah, A. (2018). Comparing Ultrasound-based Diaphragmatic Excursion with Rapid Shallow Breathing Index as a Weaning Predictor. Cureus, 10(12), e3710. https://doi.org/10.7759/cureus.3710	Wrong outcome: NIV as extubation failure
73	Kifle, N., Zewdu, D., Abebe, B., Tantu, T., Wondwosen, M., Hailu, Y., Bekele, G., & Woldetensay, M. (2022). Incidence of extubation failure and its predictors among adult patients in intensive care unit of low-resource setting: A prospective observational study. PloS one, 17(11), e0277915. https://doi.org/10.1371/journal.pone.0277915	Wrong outcome: NIV as extubation failure
74	Korupolu, R., Uhlig-Reche, H., Achilike, E. C., Reeh, C., Pedroza, C., & Stampas, A. (2022). Factors Associated With Ventilator Weaning Success and Failure in People With Spinal Cord Injury in an Acute Inpatient Rehabilitation Setting: A Retrospective Study. Topics in spinal cord injury rehabilitation, 28(2), 129–138. https://doi.org/10.46292/sci21-00062	Missing methodology: No information provided on central outcomes (extubation failure/success)
75	Kundu, R., Baidya, D., Anand, R., Maitra, S., Soni, K., & Subramanium, R. (2022). Integrated ultrasound protocol in predicting weaning success and extubation failure: a prospective observational study. Anaesthesiology intensive therapy, 54(2), 156–163. https://doi.org/10.5114/ait.2022.115351	Wrong outcome: NIV as extubation failure
76	Kuo, H. J., Chiu, H. W., Lee, C. N., Chen, T. T., Chang, C. C., & Bien, M. Y. (2015). Improvement in the Prediction of Ventilator Weaning Outcomes by an Artificial Neural Network in a Medical ICU. Respiratory care, 60(11), 1560–1569. https://doi.org/10.4187/respcare.03648	Wrong outcome: NIV as extubation failure

77	Lamia, B., Maizel, J., Ochagavia, A., Chemla, D., Osman, D., Richard, C., & Teboul, J. L. (2009). Echocardiographic diagnosis of pulmonary artery occlusion pressure elevation during weaning from mechanical ventilation. Critical care medicine, 37(5), 1696–1701. https://doi.org/10.1097/CCM.0b013e31819f13d0	Wrong outcome: Only weaning-induced PAOP investigated, no outcomes of interest
78	Le Neindre, A., Philippart, F., Luperto, M., Wormser, J., Morel-Sapene, J., Aho, S. L., Mongodi, S., Mojoli, F., & Bouhemad, B. (2021). Diagnostic accuracy of diaphragm ultrasound to predict weaning outcome: A systematic review and meta-analysis. International journal of nursing studies, 117, 103890. https://doi.org/10.1016/j.ijnurstu.2021.103890	Wrong outcome: Studies included in meta-analysis refer to NIV as extubation failure
79	Lee, C. S., Chen, N. H., Chuang, L. P., Chang, C. H., Li, L. F., Lin, S. W., & Huang, H. Y. (2017). Hypercapnic Ventilatory Response in the Weaning of Patients with Prolonged Mechanical Ventilation. Canadian respiratory journal, 2017, 7381424. https://doi.org/10.1155/2017/7381424	Wrong setting: Respiratory care center as a step-down, subacute care facility after ICU
80	Lee, K. H., Hui, K. P., Chan, T. B., Tan, W. C., & Lim, T. K. (1994). Rapid shallow breathing (frequency-tidal volume ratio) did not predict extubation outcome. Chest, 105(2), 540–543. https://doi.org/10.1378/chest.105.2.540	Wrong outcome: NIV as extubation failure
81	Leonov, Y., Kisil, I., Perlov, A., Stoichev, V., Ginzburg, Y., Nazarenko, A., & Gimelfarb, Y. (2020). Predictors of successful weaning in patients requiring extremely prolonged mechanical ventilation. Advances in respiratory medicine, 88(6), 477–484. https://doi.org/10.5603/ARM.a2020.0151	Wrong setting: Long-term ventilator facility
82	Li, S., Chen, Z., & Yan, W. (2021). Application of bedside ultrasound in predicting the outcome of weaning from mechanical ventilation in elderly patients. BMC pulmonary medicine, 21(1), 217. https://doi.org/10.1186/s12890-021-01605-4	Wrong outcome: NIV as extubation failure
83	Li, C., Li, X., Han, H., Cui, H., Wang, G., & Wang, Z. (2018). Diaphragmatic ultrasonography for predicting ventilator weaning: A meta-analysis. Medicine, 97(22), e10968. https://doi.org/10.1097/MD.0000000000010968	Wrong outcome: Studies included in meta-analysis refer to NIV as extubation failure
84	Liang, Y. R., Yang, M. C., Wu, Y. K., Tzeng, I. S., Wu, P. Y., Huang, S. Y., Lan, C. C., & Wu, C. P. (2020). Transitional Percentage of Minute Volume as a Novel Predictor of Weaning from Mechanical Ventilation in Patients with Chronic Respiratory Failure. Asian nursing research, 14(1), 30–35. https://doi.org/10.1016/j.anr.2020.01.002	Wrong setting: Respiratory care center to manage patients with prolonged mechanical ventilation but in stable hemodynamic condition
85	Lima E. J. (2013). Respiratory rate as a predictor of weaning failure from mechanical ventilation. Brazilian journal of anesthesiology (Elsevier), 63(1), 1–6. https://doi.org/10.1016/j.bjane.2012.04.001	Duplicate (was not detected in Title-/Abstract-Screening)

86	Lin, F. C., Kuo, Y. W., Jerng, J. S., & Wu, H. D. (2020). Association of weaning preparedness with extubation outcome of mechanically ventilated patients in medical intensive care units: a retrospective analysis. PeerJ, 8, e8973. https://doi.org/10.7717/peerj.8973	Wrong outcome: NIV as extubation failure
87	Liu, L., Liu, H., Yang, Y., Huang, Y., Liu, S., Beck, J., Slutsky, A. S., Sinderby, C., & Qiu, H. (2012). Neuroventilatory efficiency and extubation readiness in critically ill patients. Critical care (London, England), 16(4), R143. https://doi.org/10.1186/cc11451	Wrong outcome: NIV as extubation failure
88	Liu, J., Wang, C. J., Ran, J. H., Lin, S. H., Deng, D., Ma, Y., & Xu, F. (2021). The predictive value of brain natriuretic peptide or N-terminal pro-brain natriuretic peptide for weaning outcome in mechanical ventilation patients: Evidence from SROC. Journal of the renin-angiotensin-aldosterone system : JRAAS, 22(1), 1470320321999497. https://doi.org/10.1177/1470320321999497	Missing methodology: No information provided on central outcomes (extubation failure/success)
89	Lombardi, F. S., Cotoia, A., Petta, R., Schultz, M., Cinnella, G., & Horn, J. (2019). Prediction of extubation failure in Intensive Care Unit: systematic review of parameters investigated. Minerva anestesiologica, 85(3), 298–307. https://doi.org/10.23736/S0375-9393.18.12627-7	Wrong outcome: Studies included in meta-analysis refer to NIV as extubation failure
90	Lotfy, A., Hasanin, A., Rashad, M., Mostafa, M., Saad, D., Mahmoud, M., Hamimy, W., & Fouad, A. Z. (2021). Peripheral perfusion index as a predictor of failed weaning from mechanical ventilation. Journal of clinical monitoring and computing, 35(2), 405–412. https://doi.org/10.1007/s10877-020-00483-1	Wrong outcome: NIV as extubation failure
91	Luo, Z., Zheng, Y., Yang, L., Liu, S., Zhu, J., Zhao, N., Pang, B., Cao, Z., & Ma, Y. (2018). Neutrophil/lymphocyte ratio is helpful for predicting weaning failure: a prospective, observational cohort study. Journal of thoracic disease, 10(9), 5232–5245. https://doi.org/10.21037/jtd.2018.08.68	Wrong outcome: NIV as extubation failure
92	Maciel, L. R. M. A., Franzosi, O. S., Nunes, D. S. L., Loss, S. H., Dos Reis, A. M., Rubin, B. A., & Vieira, S. R. R. (2019). Nutritional Risk Screening 2002 Cut-Off to Identify High-Risk Is a Good Predictor of ICU Mortality in Critically Ill Patients. Nutrition in clinical practice : official publication of the American Society for Parenteral and Enteral Nutrition, 34(1), 137–141. https://doi.org/10.1002/ncp.10185	Wrong study design: Only group comparisons, no statistical calculations of probabilities
93	Mahmoodpoor, A., Fouladi, S., Ramouz, A., Shadvar, K., Ostadi, Z., & Soleimanpour, H. (2022). Diaphragm ultrasound to predict weaning outcome: systematic review and meta-analysis. Anaesthesiology intensive therapy, 54(2), 164–174. https://doi.org/10.5114/ait.2022.117273	Wrong population: Non-invasively ventilated patients are included in meta-analysis
94	Maldonado, A., Bauer, T. T., Ferrer, M., Hernandez, C., Arancibia, F., Rodriguez-Roisin, R., & Torres, A. (2000). Capnometric recirculation gas tonometry and weaning from mechanical	Wrong outcome: NIV as extubation failure

	ventilation. American journal of respiratory and critical care medicine, 161(1), 171–176. https://doi.org/10.1164/ajrccm.161.1.9904080	
95	Mallat, J., Baghdadi, F. A., Mohammad, U., Lemyze, M., Temime, J., Tronchon, L., Thevenin, D., & Fischer, M. O. (2020). Central Venous-to-Arterial PCO2 Difference and Central Venous Oxygen Saturation in the Detection of Extubation Failure in Critically Ill Patients. Critical care medicine, 48(10), 1454–1461. https://doi.org/10.1097/CCM.0000000000004446	Wrong outcome: NIV as extubation failure
96	Margetis, D., Maury, E., Boelle, P. Y., Alves, M., Galbois, A., Baudel, J. I., Offenstadt, G., Guidet, B., & Ait-Oufella, H. (2014). Peripheral microcirculatory exploration during mechanical ventilation weaning. Minerva anestesiologica, 80(11), 1188–1197.	Wrong study design: Only group comparisons, no statistical calculations of probabilities
97	Mesquida, J., Gruartmoner, G., Espinal, C., Masip, J., Sabatier, C., Villagrá, A., Gómez, H., Pinsky, M., Baigorri, F., & Artigas, A. (2020). Thenar oxygen saturation (StO2) alterations during aspontaneous breathing trial predict extubation failure. Annals of intensive care, 10(1), 54. https://doi.org/10.1186/s13613-020-00670-y	Wrong outcome: NIV as extubation failure
98	Miu, T., Joffe, A. M., Yanez, N. D., Khandelwal, N., Dagal, A. H., Deem, S., & Treggiari, M. M. (2014). Predictors of reintubation in critically ill patients. Respiratory care, 59(2), 178–185. https://doi.org/10.4187/respcare.02527	Wrong outcome: Extubation failure as the need for reintubation at any time of hospital stay
99	Montgomery, A. B., Holle, R. H., Neagley, S. R., Pierson, D. J., & Schoene, R. B. (1987). Prediction of successful ventilator weaning using airway occlusion pressure and hypercapnic challenge. Chest, 91(4), 496–499. https://doi.org/10.1378/chest.91.4.496	Wrong study design: Only group comparisons, no statistical calculations of probabilities
100	Mowafy, S. M. S., & Abdelgalel, E. F. (2018). Diaphragmatic rapid shallow breathing index for predicting weaning outcome from mechanical ventilation: Comparison with traditional rapid shallow breathing index. Egyptian Journal of Anaesthesia, 35(1), 9-17. https://doi.org/10.1016/j.egja.2018.10.003	Wrong outcome: NIV as extubation failure
101	Muhle, P., Suntrup-Krueger, S., Burkardt, K., Lapa, S., Ogawa, M., Claus, I., Labeit, B., Ahring, S., Oelenberg, S., Warnecke, T., & Dziewas, R. (2021). Standardized Endoscopic Swallowing Evaluation for Tracheostomy Decannulation in Critically Ill Neurologic Patients - a prospective evaluation. Neurological research and practice, 3(1), 26. https://doi.org/10.1186/s42466-021-00124-1	Wrong population: Patients already weaned off their MV support when being investigated
102	Nemer, S. N., & Barbas, C. S. (2011). Predictive parameters for weaning from mechanical ventilation. Jornal brasileiro de pneumologia : publicacao oficial da Sociedade Brasileira de Pneumologia e Tisilogia, 37(5), 669–679. https://doi.org/10.1590/s1806-37132011000500016	Wrong study design: Only narrative review, no new results
103	Nemer, S. N., Barbas, C. S., Caldeira, J. B., Cárias, T. C., Santos, R. G., Almeida, L. C., Azeredo, L. M., Noé, R. A., Guimarães, B. S., & Souza, P. C. (2009). A new integrative weaning index of	Wrong outcome: NIV as extubation failure

	discontinuation from mechanical ventilation. Critical care (London, England), 13(5), R152. https://doi.org/10.1186/cc8051	
104	Nikitas, G. T., Kykalos, S., Ntikoudi, E., Vasileiadis, I., Koutsoukou, A., & Nikiteas, N. I. (2022). A Novel Non-invasive Index of Cardiopulmonary Reserve for the Prediction of Failure of Weaning From Mechanical Ventilation. Cureus, 14(7), e27150. https://doi.org/10.7759/cureus.27150	Wrong outcome: NIV as extubation failure
105	Oh, J., Lim, H., Jeong, C. W., Kim, M. S., Lee, J., Kang, W. S., An, U. R., Park, J. U., Ahn, Y., Kim, Y. R., & Park, C. (2023). Clinical implication of thoracic skeletal muscle volume as a predictor of ventilation-weaning failure in brain-injured patients: A retrospective observational study. Medicine, 102(43), e35847. https://doi.org/10.1097/MD.0000000000035847	Wrong outcome: NIV as extubation failure
106	Palkar, A., Narasimhan, M., Greenberg, H., Singh, K., Koenig, S., Mayo, P., & Gottesman, E. (2018). Diaphragm Excursion-Time Index: A New Parameter Using Ultrasonography to Predict Extubation Outcome. Chest, 153(5), 1213–1220. https://doi.org/10.1016/j.chest.2018.01.007	Wrong outcome: NIV as extubation failure
107	Papaioannou, V. E., Chouvarda, I., Maglaveras, N., Dragoumanis, C., & Pneumatikos, I. (2011). Changes of heart and respiratory rate dynamics during weaning from mechanical ventilation: a study of physiologic complexity in surgical critically ill patients. Journal of critical care, 26(3), 262–272. https://doi.org/10.1016/j.jcrc.2010.07.010	Wrong outcome: NIV as extubation failure
108	Papaioannou, V. E., Chouvarda, I. G., Maglaveras, N. K., & Pneumatikos, I. A. (2011). Study of multiparameter respiratory pattern complexity in surgical critically ill patients during weaning trials. BMC physiology, 11, 2. https://doi.org/10.1186/1472-6793-11-2	Wrong outcome: NIV as extubation failure
109	Pirompanich, P., & Romsaiyut, S. (2018). Use of diaphragm thickening fraction combined with rapid shallow breathing index for predicting success of weaning from mechanical ventilator in medical patients. Journal of intensive care, 6, 6. https://doi.org/10.1186/s40560-018-0277-9	Wrong setting: Patients were enrolled not only in ICUs, but also in normal medical wards
110	Qian, Z., Yang, M., Li, L., & Chen, Y. (2018). Ultrasound assessment of diaphragmatic dysfunction as a predictor of weaning outcome from mechanical ventilation: a systematic review and meta-analysis. BMJ open, 8(9), e021189. https://doi.org/10.1136/bmjopen-2017-021189	Wrong outcome: Studies included in meta-analysis describe NIV as extubation failure
111	Robriquet, L., Georges, H., Leroy, O., Devos, P., D'escrivan, T., & Guery, B. (2006). Predictors of extubation failure in patients with chronic obstructive pulmonary disease. Journal of critical care, 21(2), 185–190. https://doi.org/10.1016/j.jcrc.2005.08.007	Wrong outcome: NIV as extubation failure
112	Sachin, S., Chakrabarti, D., Gopalakrishna, K. N., & Bharadwaj, S. (2021). Ultrasonographic evaluation of lung and heart in predicting successful weaning in mechanically ventilated neurosurgical patients. Journal of clinical monitoring and computing, 35(1), 189–197. https://doi.org/10.1007/s10877-020-00460-8	Wrong outcome: NIV as extubation failure

113	Saiphoklang, N., & Mokkongphai, N. (2021). Handgrip strength cutoff value predicting successful extubation in mechanically ventilated patients. PloS one, 16(10), e0258971. https://doi.org/10.1371/journal.pone.0258971	Wrong outcome: NIV as extubation failure
114	Saiphoklang, N., & Tepwimonpetkun, C. (2020). Interest of hand grip strength to predict outcome in mechanically ventilated patients. Heart & lung : the journal of critical care, 49(5), 637–640. https://doi.org/10.1016/j.hrtlng.2020.03.019	Wrong study design: Only group comparisons, no statistical calculations of probabilities
115	Sanson, G., Sartori, M., Dreas, L., Ciraolo, R., & Fabiani, A. (2018). Predictors of extubation failure after open-chest cardiac surgery based on routinely collected data. The importance of a shared interprofessional clinical assessment. European journal of cardiovascular nursing, 17(8), 751–759. https://doi.org/10.1177/1474515118782103	Wrong outcome: NIV as extubation failure
116	Saravanan, R., Nivedita, K., Karthik, K., & Venkatraman, R. (2022). Role of diaphragm ultrasound in weaning mechanically ventilated patients: A prospective observational study. Indian journal of anaesthesia, 66(8), 591–598. https://doi.org/10.4103/ija.ija_229_22	Wrong outcome: NIV as extubation failure
117	Sassoon, C. S., & Mahutte, C. K. (1993). Airway occlusion pressure and breathing pattern as predictors of weaning outcome. The American review of respiratory disease, 148(4 Pt 1), 860–866. https://doi.org/10.1164/ajrccm/148.4_Pt_1.860	Wrong outcome: Respiratory failure as an outcome until 48h of extubation, but no reintubation or death as recommended by our underlying definition (Beduneau et al., 2017).
118	Savla, P., Toor, H., Podkovik, S., Mak, J., Kal, S., Soliman, C., Ku, A., Majeed, G., & Miulli, D. E. (2021). A Reassessment of Weaning Parameters in Patients With Spontaneous Intracerebral Hemorrhage. Cureus, 13(1), e12539. https://doi.org/10.7759/cureus.12539	Missing methodology: No information provided on central outcomes (extubation failure/success)
119	Schifelbain, L. M., Vieira, S. R., Brauner, J. S., Pacheco, D. M., & Naujorks, A. A. (2011). Echocardiographic evaluation during weaning from mechanical ventilation. Clinics (Sao Paulo, Brazil), 66(1), 107–111. https://doi.org/10.1590/s1807-59322011000100019	Wrong study design: Only group comparisons, no regression analysis
120	Serrano, N., García, C., Villegas, J., Huidobro, S., Henry, C. C., Santacreu, R., Mora, M. L., & Epidemiological Project for ICU Research and Evaluation (EPICURE) (2005). Prolonged intubation rates after coronary artery bypass surgery and ICU risk stratification score. Chest, 128(2), 595–601. https://doi.org/10.1378/chest.128.2.595	Wrong outcome: Extubation failure is defined as remaining on intubated
121	Shamil, P. K., Gupta, N. K., Ish, P., Sen, M. K., Kumar, R., Chakrabarti, S., & Gupta, N. (2022). Prediction of Weaning Outcome from Mechanical Ventilation Using Diaphragmatic Rapid Shallow Breathing Index. Indian journal of critical care medicine : peer-reviewed, official publication of Indian Society of Critical Care Medicine, 26(9), 1000–1005. https://doi.org/10.5005/jp-journals-10071-24316	Wrong outcome: NIV as extubation failure

122	Sharma, A., Karna, S. T., Tandon, M., Pandey, C. K., Chaturvedi, R., Vyas, V., & Goel, A. D. (2018). Use of ultrasound-guided preoperative diaphragmatic thickness as a predictor of postoperative weaning failure in recipients and donors scheduled for living donor liver transplant surgery. Saudi journal of anaesthesia, 12(3), 406–411. https://doi.org/10.4103/sja.SJA_12_18	Wrong setting: Assessment was conducted preoperative, not in the ICU
123	Shin, S. H., Heath, K., Reed, S., Collins, J., Weireter, L. J., & Britt, L. D. (2008). The cuff leak test is not predictive of successful extubation. The American surgeon, 74(12), 1182–1185. https://doi.org/10.1177/000313480807401210	Wrong outcome: Extubation failure is defined as ventilator length of stay and postextubation stridor and therefore not in line with our definition (according to Béduneau 2017).
124	Solsona, J. F., Díaz, Y., Vázquez, A., Pilar Gracia, M., Zapatero, A., & Marrugat, J. (2009). A pilot study of a new test to predict extubation failure. Critical care (London, England), 13(2), R56. https://doi.org/10.1186/cc7783	Wrong outcome: NIV as extubation failure
125	Song, J., Qian, Z., Zhang, H., Wang, M., Yu, Y., Ye, C., Hu, W., & Gong, S. (2022). Diaphragmatic ultrasonography-based rapid shallow breathing index for predicting weaning outcome during a pressure support ventilation spontaneous breathing trial. BMC pulmonary medicine, 22(1), 337. https://doi.org/10.1186/s12890-022-02133-5	Wrong outcome: NIV as extubation failure
126	Song, J., Luo, Q., Lai, X., Hu, W., Yu, Y., Wang, M., Yang, K., Chen, G., Chen, W., Li, Q., Hu, C., & Gong, S. (2024). Combined cardiac, lung, and diaphragm ultrasound for predicting weaning failure during spontaneous breathing trial. Annals of intensive care, 14(1), 60. https://doi.org/10.1186/s13613-024-01294-2	Wrong outcome: NIV as extubation failure
127	Spadaro, S., Grasso, S., Mauri, T., Dalla Corte, F., Alvisi, V., Ragazzi, R., Cricca, V., Biondi, G., Di Mussi, R., Marangoni, E., & Volta, C. A. (2016). Can diaphragmatic ultrasonography performed during the T-tube trial predict weaning failure? The role of diaphragmatic rapid shallow breathing index. Critical care (London, England), 20(1), 305. https://doi.org/10.1186/s13054-016-1479-y	Wrong outcome: NIV as extubation failure
128	Su, W. L., Chen, Y. H., Chen, C. W., Yang, S. H., Su, C. L., Perng, W. C., Wu, C. P., & Chen, J. H. (2010). Involuntary cough strength and extubation outcomes for patients in an ICU. Chest, 137(4), 777–782. https://doi.org/10.1378/chest.07-2808	Missing methodology: No information provided on central outcomes (extubation failure/success)
129	Suraseranivong, R., Krairit, O., Theerawit, P., & Sutherasan, Y. (2018). Association between age-related factors and extubation failure in elderly patients. PloS one, 13(11), e0207628. https://doi.org/10.1371/journal.pone.0207628	Wrong study design: Only explanatory but no predictive factors were investigated (according to our underlying definition)
130	Teixeira, C., Teixeira, P. J., de Leon, P. P., & Oliveira, E. S. (2009). Work of breathing during successful spontaneous breathing trial. Journal of critical care, 24(4), 508–514. https://doi.org/10.1016/j.jcrc.2008.10.013	Wrong outcome: NIV as extubation failure

131	Teixeira, C., Zimermann Teixeira, P. J., Hohër, J. A., de Leon, P. P., Brodt, S. F., & da Siva Moreira, J. (2008). Serial measurements of f/VT can predict extubation failure in patients with f/VT < or = 105?. Journal of critical care, 23(4), 572–576. https://doi.org/10.1016/j.jcrc.2007.12.011	Wrong outcome: NIV as extubation failure
132	Tenza-Lozano, E., Llamas-Alvarez, A., Jaimez-Navarro, E., & Fernández-Sánchez, J. (2018). Lung and diaphragm ultrasound as predictors of success in weaning from mechanical ventilation. Critical ultrasound journal, 10(1), 12. https://doi.org/10.1186/s13089-018-0094-3	Wrong outcome: NIV as extubation failure
133	Thabet, D. B., Makhlouf, H. A., Hasan, A. A., Mekkawy, A. I., & Ghanem, M. K. (2021). Serial ultrasonographic monitoring of diaphragmatic and mid-upper arm muscle thickness in mechanically ventilated respiratory patients: A single-center observational study. The clinical respiratory journal, 15(1), 11–18. https://doi.org/10.1111/crj.13263	Wrong outcome: NIV as extubation failure
134	Theerawit, P., Eksombatchai, D., Sutherasan, Y., Suwatanapongched, T., Kiatboonsri, C., & Kiatboonsri, S. (2018). Diaphragmatic parameters by ultrasonography for predicting weaning outcomes. BMC pulmonary medicine, 18(1), 175. https://doi.org/10.1186/s12890-018-0739-9	Wrong outcome: NIV as extubation failure
135	Torrini, F., Gendreau, S., Morel, J., Carteaux, G., Thille, A. W., Antonelli, M., & Mekontso Dessap, A. (2021). Prediction of extubation outcome in critically ill patients: a systematic review and meta-analysis. Critical care (London, England), 25(1), 391. https://doi.org/10.1186/s13054-021-03802-3	Wrong outcome: Studies included in meta-analysis describe NIV as extubation failure
136	Vargas, F., Boyer, A., Bui, H. N., Salmi, L. R., Guenard, H., Gruson, D., & Hilbert, G. (2008). Respiratory failure in chronic obstructive pulmonary disease after extubation: value of expiratory flow limitation and airway occlusion pressure after 0.1 second (P0.1). Journal of critical care, 23(4), 577–584. https://doi.org/10.1016/j.jcrc.2007.12.009	Wrong outcome: Prediction of respiratory failure was investigated
137	Vivier, E., Muller, M., Putegnat, J. B., Steyer, J., Barrau, S., Boissier, F., Bourdin, G., Mekontso-Dessap, A., Levrat, A., Pommier, C., & Thille, A. W. (2019). Inability of Diaphragm Ultrasound to Predict Extubation Failure: A Multicenter Study. Chest, 155(6), 1131–1139. https://doi.org/10.1016/j.chest.2019.03.004	Wrong study design: Only group comparisons, no statistical calculations of probabilities
138	Wan, Y. F., Zheng, Y. L., Du, Y. P., Huai, D., Peng, Y. G., Niu, H. Y., Xu, C. Q., Shi, Y., & Zheng, D. H. (2015). Utility of uric acid as a risk marker of extubation success in chronic obstructive pulmonary disease. Clinical laboratory, 61(3-4), 337–344. https://doi.org/10.7754/clin.lab.2014.140721	Wrong outcome: NIV as extubation failure
139	Wang, S., Zhang, L., Huang, K., Lin, Z., Qiao, W., & Pan, S. (2014). Predictors of extubation failure in neurocritical patients identified by a systematic review and meta-analysis. PloS one, 9(12), e112198. https://doi.org/10.1371/journal.pone.0112198	Wrong outcome: Studies included in meta-analysis describe NIV as extubation failure

140	Wang, Y., Yi, Y., Zhang, F., Yao, Y. Y., Chen, Y. X., Wu, C. M., Wang, R. Y., & Yan, M. (2024). Lung Ultrasound Score as a Predictor of Failure to Wean COVID-19 Elderly Patients off Mechanical Ventilation: A Prospective Observational Study. Clinical interventions in aging, 19, 313–322. https://doi.org/10.2147/CIA.S438714	Wrong outcome: NIV as extubation failure
141	Welte, T. M., Gabriel, M., Hopfengärtner, R., Rampp, S., Gollwitzer, S., Lang, J. D., Stritzelberger, J., Reindl, C., Madžar, D., Sprügel, M. I., Huttner, H. B., Kuramatsu, J. B., Schwab, S., & Hamer, H. M. (2022). Quantitative EEG may predict weaning failure in ventilated patients on the neurological intensive care unit. Scientific reports, 12(1), 7293. https://doi.org/10.1038/s41598-022-11196-7	Wrong outcome: NIV as extubation failure
142	Wu, C., Hu, L., Shen, Q., Xu, H., & Huang, H. (2023). Predictive value of extubation failure by decrease in central venous oxygen saturation: A systematic review and meta-analysis. Heliyon, 9(7), e18227. https://doi.org/10.1016/j.heliyon.2023.e18227	Wrong outcome: Studies included in meta-analysis describe NIV as extubation failure
143	Xu, X., Wu, R., Zhang, Y. J., Li, H. W., He, X. H., & Wang, S. M. (2020). Value of Combination of Heart, Lung, and Diaphragm Ultrasound in Predicting Weaning Outcome of Mechanical Ventilation. Medical science monitor : international medical journal of experimental and clinical research, 26, e924885. https://doi.org/10.12659/MSM.924885	Wrong outcome: NIV as extubation failure
144	Yan, Y., Luo, J., Wang, Y., Chen, X., Du, Z., Xie, Y., & Li, X. (2022). Development and validation of a mechanical power-oriented prediction model of weaning failure in mechanically ventilated patients: a retrospective cohort study. BMJ open, 12(12), e066894. https://doi.org/10.1136/bmjopen-2022-066894	Wrong outcome: NIV as extubation failure
145	Yan, Y., Xie, Y., Chen, X., Sun, Y., Du, Z., Wang, Y., & Li, X. (2022). Mechanical power is associated with weaning outcome in critically ill mechanically ventilated patients. Scientific reports, 12(1), 19634. https://doi.org/10.1038/s41598-022-21609-2	Wrong outcome: NIV as extubation failure
146	Yang, H., Ni, Y., Huang, D., & Liang, Z. (2023). Ventilatory ratio as a predictor for extubation failure in critical ill patients based on MIMIC-IV database (from 2008 to 2019). Frontiers in physiology, 14, 1137115. https://doi.org/10.3389/fphys.2023.1137115	Wrong outcome: NIV as extubation failure
147	Yoo, J. W., Lee, S. J., Lee, J. D., & Kim, H. C. (2018). Comparison of clinical utility between diaphragm excursion and thickening change using ultrasonography to predict extubation success. The Korean journal of internal medicine, 33(2), 331–339. https://doi.org/10.3904/kjim.2016.152	Wrong outcome: NIV as extubation failure
148	Zambon, M., Greco, M., Bocchino, S., Cabrini, L., Beccaria, P. F., & Zangrillo, A. (2017). Assessment of diaphragmatic dysfunction in the critically ill patient with ultrasound: a	Missing methodology: No information provided on central outcomes (extubation failure/success)

	systematic review. Intensive care medicine, 43(1), 29–38. https://doi.org/10.1007/s00134-016-4524-z	
149	Zanotti, E., Rubini, F., Iotti, G., Braschi, A., Palo, A., Bruschi, C., Fracchia, C., & Nava, S. (1995). Elevated static compliance of the total respiratory system: early predictor of weaning unsuccess in severed COPD patients mechanically ventilated. Intensive care medicine, 21(5), 399–405. https://doi.org/10.1007/BF01707408	Wrong outcome: Weaning failure lasts until 4 months after disconnection
150	Zapata, L., Vera, P., Roglan, A., Gich, I., Ordonez-Llanos, J., & Betbesé, A. J. (2011). B-type natriuretic peptides for prediction and diagnosis of weaning failure from cardiac origin. Intensive care medicine, 37(3), 477–485. https://doi.org/10.1007/s00134-010-2101-4	Wrong scope: Investigation of predictors of heart failure, no investigation of predictors of weaning outcome
151	Zeng, Z., Tang, X., Liu, Y., He, Z., & Gong, X. (2022). Interpretable recurrent neural network models for dynamic prediction of the extubation failure risk in patients with invasive mechanical ventilation in the intensive care unit. BioData mining, 15(1), 21. https://doi.org/10.1186/s13040-022-00309-7	Wrong outcome: NIV as extubation failure
152	Zhang, X., Yuan, J., Zhan, Y., Wu, J., Liu, B., Zhang, P., Yu, T., Wang, Z., Jiang, X., & Lu, W. (2020). Evaluation of diaphragm ultrasound in predicting extubation outcome in mechanically ventilated patients with COPD. Irish journal of medical science, 189(2), 661–668. https://doi.org/10.1007/s11845-019-02117-1	Wrong outcome: NIV as extubation failure
153	Zhang, Z., Tang, W., Ren, Y., Zhao, Y., You, J., Wang, H., Zhao, S., & Zuo, X. (2024). Prediction of ventilator weaning failure in postoperative cardiac surgery patients using vasoactive-ventilation-renal score and nomogram analysis. Frontiers in cardiovascular medicine, 11, 1364211. https://doi.org/10.3389/fcvm.2024.1364211	Wrong outcome: NIV as extubation failure
154	Zhao, Q. Y., Wang, H., Luo, J. C., Luo, M. H., Liu, L. P., Yu, S. J., Liu, K., Zhang, Y. J., Sun, P., Tu, G. W., & Luo, Z. (2021). Development and Validation of a Machine-Learning Model for Prediction of Extubation Failure in Intensive Care Units. *Frontiers in medicine*, *8*, 676343. https://doi.org/10.3389/fmed.2021.676343	Wrong outcome: NIV as extubation failure
