# Supplementary material for: Predictors of weaning failure in ventilated intensive care patients: a systematic evidence map
Source: Crit Care. 2024 Nov 12;28:366. doi: 10.1186/s13054-024-05135-3 (PMC11556093; doi:10.1186/s13054-024-05135-3)
Supplement: Supplementary file 3 — Additional file3 (PDF 392 KB) This supplement contains the study characteristics table and further information extracted from the 140 included original studies [file 13054_2024_5135_MOESM3_ESM.pdf]

Additional file 3: Characteristics of included studies

Systematic evidence map on predictors of weaning failure

Abbreviations:

aOR = adjusted odds ratio; *AUC* = area under the curve; *CPF* = Cough peak flow; *CROP* = compliance, rate, oxygenation, pressure index; *DE* = Diaphragm excursion; *DF* = Decannulation Failure; *DS* = Decannulation success; *DTF* = Diaphragm thickening fraction; *EF* = extubation failure; *ES* = extubation success; FiO_2 = fraction of inspired oxygen; *GCS* = Glasgow coma score; *Hb* = Hemoglobin; *HR* = Heart rate; *ICU* = Intensive care unit; *LUS* = Lung ultrasound; *MAP* = Mean Airway Pressure; *MIP* = Maximal inspiratory pressure; *OR* = odds ratio; *PCEF* = Peak Cough exspiratrory flow; *PSV* = Pressure support ventilation; *P/F ratio* = PaO_2/FiO_2 (Horovitz-Index); *Rel R.* = Relative risk; *RISC* = Re-Intubation Scale Calculation; *ROX index* = ratio of SpO2/FiO2 to RR; *RR* = Respiratory rate; *RSBI* = Rapid shallow breathing index (RR/V_t); *SBT* = spontaneous breathing trial; *SpO2* = Saturation of peripheral oxygen; V_t = Tidal volume; V_e = Minute ventilation; *WF* = weaning failure; *WS* = weaning success

Author, year, country	Study design	Population	Setting	Predictive factor(s)	Outcome(s)	Result(s)	DOI
Abplanalp, 2023, USA	Retrospective data analysis	2,263 patients (1,989 ES, 274 EF)	ICUs in 8 hospitals	Respiratory system compliance (RC), RSBI, BMI, P/F ratio, SOFA score	Extubation failure (reintubation within 72h)	RC aOR = 1.45 (24h), 1.58 (72h); BMI aOR = 0.98 (24h), 0.99 (72h); P/F aOR = 1.00 (24h), 0.98 (72h); SOFA score aOR = 1.02 (24h), 1.02 (72h); RSBI aOR = 1.00 (24h), 1.00 (72h)	10.1007/s00408-023-00625-7
Alam, 2022, Bangladesh	Prospective observational study	31 patients (18 ES, 13 EF)	ICU at a tertiary hospital	DE, DTF, RSBI	Extubation failure (reintubation within 48h)	AUC for DE = 0.83; AUC for DTF = 0.69; AUC for RSBI = 0.58	10.4266/acc.2021.01354
Andrade Filho, 2021, Brazil	Prospective observational cohort study	69 Covid-19 patients (15,3% EF)	ICU	ROX index, SpO2/FiO2, Hb, Mean blood pressure	Extubation failure (reintubation within 48h)	Mbp OR = 0.85 (n.s.); Hb OR = 1.01 (n.s.); SpO2/FiO2 OR = 0.95; ROX OR = 0.45	10.4187/respcare.08564
Antonio, 2018, Brazil	Prospective multicenter observational study	250 patients (51 SBT failure)	2 medical-surgical ICUs	B-Lines in LUS	SBT failure	B-Line sensitivity = 0.47; specificity = 0.64; positive predictive value = 0.25; negative predictive value = 0.82	10.4187/respcare.05817
Antonio, 2015, Brazil	Prospective observational study	250 patients (51 SBT failure)	2 medical-surgical ICUs	Fluid Balance	SBT failure	No overall group differences, no log. reg.; Subgroup COPD: AUC for 48h fluid balance in COPD patients = 0.70	10.4187/respcare.03172

Antonio, 2017, Brazil	Prospective observational cohort study	170 patients (28 SBT failure)	adult medical-surgical ICU	Radiographic score (RS)	SBT failure	AUC for RS = 0.58	10.1590/S1806-37562016000000360
Arcanjo, 2023, Brazil	Retrospective case-control study	480 patients (415 ES, 65 EF)	2 ICUs	Fluid Balance (24h before extubation), amount of pulmonary secretion (physical therapist notes), cough effectiveness (ineffective when oro-tracheal aspiration was needed after extubation)	Extubation failure (reintubation within 48h)	Hydric balance OR = 1.00; Ineffective cough OR = 6.588	10.1590/1518-8345.6224.3864
Asehnoune, 2017, France	Prospective observational cohort study	437 patients with brain injury (338 ES, 99 EF)	4 ICUs	Visage Score (Age <40, Visual pursuit, swallowing attempts, gcs > 10); Visual pursuit; Swallowing attempts; GCS	Extubation failre (reintubation within 48h)	AUC for Visage Score = 0.75; Visual pursuit OR = 2.79; Swallowing attempts OR = 2.9; GCS > 10 OR = 2.4	10.1097/ALN.0000000000001725
Asmita, 2022, India	RCT	100 patients (79 ES, 21 EF)	ICU	DTF% = ((Dti-Dte)/Dte)x100; CI = Composite Score(DTF% Score + RSBI Score)	Extubation failure (reintubation within 48h)	DTF% AUC = 0.977	10.7860/JCDR/2022/54807.16519
Banerjee, 2018, India	Prospective observational study	53 patients (40 ES, 13 EF)	Critical care unit	RSBI, DE, DTF, DC (diaphragmatic contractioin), LUS score	Extubation failure (reintubation within 48h)	AUC for RSBI = 0,99; AUC for DC = 0,93; AUC for DE = 0,86; AUC for DTF = 0,73; AUC for LUS = 0,78	10.4103/ijccm.IJCCM_331_17
Bansal, 2022, USA	Retrospective cohort study	6,161 patients (5,415 ES, 746 EF)	ICU	"RISC score (comprised of: underweight status (BMI < 18.5 kg/m2), GCS score ≥ 10, MAP closest to 1 min after SBT start within 15 min < 10 cmH2O, fluid balance of ≥ 1,500 mL 24 h prior to extubation, mechanical ventilation ≥ 5 days); GCS, Mean airway pressure, Fluid balance"	Extubation failure (reintubation within 72h)	AUC for RISC score = 0.67; GCS OR = 1.68; MAP OR = 1.71; Fluid balance OR = 2.30	10.3389/fmed.2021.789440
Beuret, 2009, France	Prospective observational study	130 patients (116 ES, 14 EF)	ICU	PCEF (> 35 l/min)	Extubation failure (reintubation within 48h)	PCEF Rel. R = 6.9	10.1007/s00134-009-1404-9
Bilello, 2013, USA	Retrospective data analysis	173 Extubations in 163 blunt trauma patients (147 ES, 26 EF)	Level 1 Trauma Center	Alveolar-arterial oxygen difference (A-a), P/F ratio	Extubation failure (reintubation within 72h)	AUC for P/F = 0.8; P/F ratio OR = 8.7; AUC for A-a = 0.8; A-a OR = 9.2	10.1097/TA.0b013e3182946649

Blumhof, 2016, USA	Prospective observational study	33 patients (26 ES, 7 EF)	ICU	Percent change in diaphragmatic thickness (Δtdi%)	Extubation failure (reintubation within 48h)	AUC for Δtdi% at PS niveaus 5/5, 10/5 and 15/5 = 0,86; 0,82 and 0,66	10.1007/s00408-016-9911-2
Boniatti, 2014, Brazil	Prospective observational study	153 patients (130 ES, 23 EF)	ICU	Modified integrative weaning index (IWI)	Extubation failure (reintubation within 48h)	Modified IWI was no accurate predictor of EF; AUC results are missing in the article.	10.4187/respcare.02652
Bouachour, 1996, France	Prospective observational study	26 COPD patients (20 ES, 6 WF)	ICU	pHim (gastric intramucosal pH), pCO2im (intramucosal pCO2), Vt, RSBI, PaO2/PAO2	Weaning failure (SBT failure or extubation failure [reintubation within 24h])	AUC for Vt = 0.62; AUC for RSBI = 0.62; AUC for PaO2/PAO2 = 0.49; AUC for pCO2im = 0.85; AUC for pHim = 0.91	10.1183/09031936.96.09091868
Capdevila, 1995, France	Prospective observational study	67 patients (55 ES, 12 EF)	ICU	P0.1/MIP ratio, P0.1 (airway occlusion pressure), RSBI, MIP (maximal inspiratory pressure)	Extubation failure (reintubation within 48h)	AUC for MIP = 0.71; AUC for P0.1 = 0.93; AUC for P0.1/MIP = 0.99; AUC for RSBI = 0.72	10.1378/chest.108.2.482
Chaudhuri, 2022, India	Prospective observational study	120 patients (83 ES, 37 WF)	ICU	HACOR Score (heart rate, acidosis, consciousness level, oxygenation and respiratory rate), SOFA score, CCI (Charlson comorbidity index)	Weaning failure (SBT failure or extubation failure [reintubation within 48h])	AUC for HACOR = 0.95; SOFA OR = 2.82 (n.s.); CCI OR = 1.0 (n.s.)	10.5005/jp-journals-10071-24280
Chen, 2020, Taiwan	Prospective observatinal cohort study	92 patients (20 EF)	Medical ICU	FRC (Functional residual capacity), FRC/pBW (predicted body weight), RSBI	Extubation failure (reintubation within 48h)	FRC OR = 1.14; AUC for FRC/pBW = 0.77; AUC for RSBI = 0.70	10.1038/s41598-020-58008-4
Chien, 2008, Taiwan	Prospective observational study	90 patients (71 ES, 19 EF)	ICU	Percentage change of BNP (B-type natriuretic peptide) levels during 2h SBT	Extubation failure (reintubation within 48h)	BNP change during SBT OR = 1.19	10.1097/CCM.0b013e31816f49ac
Chuang, 2023, Taiwan	Retrospective cohort study	268 patients (19 EF)	ICU	BMI (Body mass index), MIP (maximal inspiratory pressure), GCS, Cuff-leak, RSBI	Extubation failure (reintubation within 72h)	BMI (underweight vs. normal) OR = 3.80; BMI (overweight vs. normal) OR = 0.54 (n.s.); GCS OR = 0.89 (n.s.); Cuff-leak OR = 2.28 (n.s.); RSBI OR = 1.16 (n.s.); MIP OR = 1.04	10.1371/journal.pone.0284564
Cinotti, 2022, International	Prospective observational multicenter study	1,512 neurocritical care patients (319 Tracheostomies, 962 ES, 231 EF)	73 ICUs in 18 countries	ENIO Score: complete score, simplified score (TBI, vigorous cogh, gag reflex, swallowing attempts, endotracheal suctioning <= 2 times per hour, GCS motor score = 6, body	Extubation failure (reintubation within 5 days)	AUC for ENIO score = 0.71	10.1007/s00134-022-06825-8

				temperature the day of extubation)			
da Silva Guimaraes, 2019, Brazil	Prospective observational study	70 patients (49 WS, 21 WF)	ICU	Timed inspiratory effort (TIE) index	Extubation failure (reintubation within 48h)	AUC for TIE = 0.93	10.4187/respcare.06367
Danaga, 2009, Brazil	Prospective observational study	73 patients (58 ES, 15 EF)	ICU	RSBI during T-piece SBT	Extubation failure (reintubation within 48h)	AUC for RSBI = 0.78	10.1590/s1806-37132009000600007
Decavele, 2022, France	Prospective observational study	58 patients (25 SBT success, 33 SBT failure)	ICU	MV-RDOS (Mechanical Ventilation—Respiratory Distress Observation Scale), RSBI	SBT Failure	AUC for 15 min MV-RDOS = 0.804; AUC for 15 min RSBI = 0.789	10.1186/s13054-022-04028-7
DeHaven, 1996, USA	Prospective observational study	589 patients (543 ES, 46 EF)	Trauma ICU	RR	Extubation failure (reintubation within 72h)	Diagnostic accuracy of RR = 0.77	10.1097/00003246-199606000-00017
dos Reis, 2013, Brazil	Prospective observational cohort study	119 traumatic brain injury patients (104 ES, 15 EF)	ICU	RSBI	Extubation failure (reintubation within 48h)	AUC for RSBI = 0.64	10.5935/0103-507X.20130037
dos Reis, 2017, Brazil	Prospective observational study	311 traumatic brain injury patients (268 ES, 43 EF)	ICU	Risk score (consisting of: sex, GCS <=5, secretion volume, absent or weak cough, mechanical ventilation >= 10 days), cough strength, secretion volume, motor GCS	Extubation failure (reintubation within 48h)	Motor GCS OR = 4.89; Secretion volume OR = 3.96; Cough strength OR = 3.03	10.1016/j.jcrc.2017.07.051
Dres, 2018, France	Retrospective data analysis	76 patients (28 SBT failure)	ICU	Ptr,stim (Twitch trachael pressure in response to magnetic phrenic stimulation); DTF	SBT Failure	AUC for Ptr,stim = 0.80; AUC for DTF = 0.82	10.1186/s13613-018-0401-y
Dres, 2021, France	Prospective observational study	122 patients (101 ES, 21 EF)	Multicenter ICU (1 France, 1 Canada)	Dyspnoea-VAS (Dyspnoea visual analogue scale), IC-RDOS (Intensive Care Respiratory Distress Observational Scale), thickening fraction of parasternal intercostal (TFic) and diaphragm (TFdi), TFic/TFdi, muscle strength MRC Score (Medical Research Council)	Extubation failure (reintubation or death within 7 days)	AUC for Dyspnoe-VAS = 0.78; AUC for IC-RDOS = 0.74; AUC for MRC score = 0.69; AUC for TFic = 0.81; AUC for DTF = 0.73; AUC for TFic/DTF = 0.81	10.1183/13993003.00002-2021

Dubo, 2021, Chile, Ecuador	Prospective observational multicenter study	165 patients (154 ES, 11 EF)	4 medical-surgical ICUs in Chile and Ecuador	CVP (Central Venous Pressure) at two minutes SBT, dCVP (CVP change from baseline), HR, RR, ScvO2	Extubation failure (reintubation within 48h)	dCVP OR = 1.70; ScvO2 OR = 0.99 (n.s.); HR OR = 0.99 (n.s.); RR OR = 0.99 (n.s.)	10.1371/journal.pone.0225181
Eksombatchai, 2023, Thailand	Prospective cross-sectional study	130 patients (122 ES, 8 EF)	ICU	RR/DTF (Respiratory rate to diaphragm thickening fraction ratio), diaphragmatic inspiratory excursion (DE), RR/DE, time to peak inspiratory amplitude of the diaphragm (TPIA), DTF, VC (Vital Capacity), MIP, RSBI	Extubation failure (reintubation within 48h)	AUC for right RR/DTF = 0.762; AUC for left RR/DTF = 0.746; AUC for righ RR/DE = 0.737; AUC for left RR/DE = 0.701; AUC for right DE = 0.730; AUC for right DTF = 0.775; AUC for left DTF = 0.725; AUC for RSBI = 0.692; AUC for VC = 0.732	10.1186/s12890-023-02392-w
El Khoury, 2009, USA	Retrospective study	154 patients (142 ES, 12 EF)	Medical ICUs	P/F-Ratio (PaO2/FiO2), RSBI	Extubation failure (reintubation within 48h)	AUC for RSBI = 0.50; AUC for P/F-ratio = 0.62	10.1016/j.hrtlng.2009.10.020
El-Baradey, 2015, Egypt	RCT	120 patients (108 ES, 12 EF)	ICU	Integrative weaning index (IWI)	Extubation failure (reintubation within 48h)	Diagnostic accuracy of IWI = 0.92	10.4103/0972-5229.171352
Elshazly, 2020, Egypt	Prospective observational study	62 patients (34 ES, 28 EF)	ICU	DE, DTF	Extubation failure (reintubation within 48h)	AUC results for DE and DTF are missing in article; DE sensitivity = 97 %; DE specificity = 82 %; DTF sensitivity = 91.2 %; DTF specificity = 60.7 %	10.4046/trd.2020.0045
Epstein, 1995, USA	Prospective observational study	94 patients (76 ES, 18 EF)	Medical ICU	RSBI	Extubation failure (reintubation or death within 72h)	RSBI (cut-off 100) sensitivity = 0.92; RSBI specificity = 0.22; RSBI PPV = 0.93; RSBI NPV = 0.40	10.1164/ajrccm.152.2.7633705
Er, 2021, Turkey	Prospective study	38 patients (23 ES, 15 EF)	Medical ICU	Trf (thickness of rectus femoris muscle), Tvi (thickness of vastus intermedius muscle), Trf+Tvi, DTF, DE, CFS (Clinical Frailty Scale)	Extubation failure (reintubation or death within 7 days)	AUC for Trf+vi = 0.71; AUC for DE = 0.74; BMI OR = 1.1 (n.s.); CFS OR = 0.8 (n.s.); No difference in DTF	10.1016/j.rmed.2021.106503
Fabregat, 2021, Spain	Retrospective observational study	697 "programmed" extubations (50 EF)	ICU	ML classifiers: SVM with radial basis, GBM with Bernoulli loss, and Linear Discriminant Analysis (LDA) (trained on HR, RR, Vt, Pip, Pplat, SpO2/FiO2, ROX, RSBI, GCS, RASS, Apache II, BMI)	Extubation failure (reintubation within 48h)	AUC for SVM = 0.983; AUC for GBM = 0.961; AUC for LDA = 0.794	10.1016/j.cmpb.2020.105869
Farghaly, 2015, Egypt	Prospective observational study	30 patients (16 ES, 14 EF)	Respiratory ICU	BNP2 (Brain natriuretic peptide after 2h SBT), BNP% (percent change	Weaning failure (SBT failure or extubation	AUC for BNP2 = 0.569; AUC for BNP% = 0.796; PaCO2 OR = 1.249 (n.s.)	10.1016/j.aucc.2014.12.002

				in the BNP level during the 2-h SBT), PaCO2	failure [reintubation within 48h])		
Fathy, 2020, Egypt	Prospective observational study	64 patients (41 ES, 23 EF)	Surgical ICU	TFC (Thoracic fluid content (measured using electrical cardiometry device)), HCO3, P/F ratio, RSBI	Extubation failure (reintubation within 48h)	AUC for TFC = 0.69; AUC for HCO3 = 0.60; AUC for P/F ratio = 0.64; AUC for RSBI = 0.51	10.1186/s40560-020-00439-2
Fazio, 2023, USA	Prospective observational study	61 patients (55 ES, 6 EF)	6 ICUs	Total WOB (total work of breathing in Proportional assist ventilation (PAV))	Extubation failure (reintubation within 72h)	AUC for WOB = 0.85	10.4187/respcare.10225
Fernandez, 2004, Spain	Prospective observational multicenter study	114 patients (93 ES, 21 EF)	ICU	P0.1 (the pressure in the first 100 ms of an occluded inspiration), RSBI, P0.1*RSBI	Extubation failure (reintubation within 48h)	AUC for RSBI = 0.53; AUC for P0.1 = 0.59; AUC for P0.1*RSBI = 0.61	10.1007/s00134-003-2070-y
Fontela, 2021, Brazil	Prospective observational study	102 patients (72 SBT success, 30 SBT failure)	3 general ICUs	MRC Score (Medical Research Council (measuring Muscle Strength)), Handgrip Strength	SBT failure	MRC Score OR = 0.91 (multivariate); Handgrip strength OR = 0.88 (univariate)	10.4187/respcare.07739
Fossat, 2022, France	Prospective observational study	100 patients (86 ES, 14 EF)	ICU	DE, RSBI, D-RSBI (Diaphragmatic RSBI), RSDI (Rapid Shallow diaphragmatic index), DTF, UDL (Ultrasound Diaphragmatic Load: (RR x DE^3)/DTF)), URML (Ultrasound Respiratory Muscles Load: (RR x DE^3)/(DTF + accessory muscle activity)	Extubation failure (reintubation with 7 days)	At 5 minutes of SBT: AUC for RSBI = 0.53; AUC for RSDI = 0.54; AUC for D-RSBI = 0.56; AUC for DE = 0.59. At 25 minutes of SBT: AUC for RSBI = 0.54; AUC for RSDI = 0.55; AUC for D-RSBI = 0.56; AUC for DE = 0.56. All patients assessed for DTF: AUC for RSBI = 0.57; AUC for RSDI = 0.65; AUC for D-RSBI = 0.66; AUC for DTF = 0.58; AUC for UDL = 0.72; AUC for URML = 0.77	10.1016/j.aucc.2021.05.008
Frutos-Vivar, 2006, International	Prospective observational multicenter study	900 (779 ES, 121 EF)	ICUs in 37 hospitals in 8 countries	RSBI, Fluid Balance (24h)	Extubation failure (reintubation within 72h)	RSBI OR = 1.009; Fluid balance OR = 1.70	10.1378/chest.130.6.1664
Fukuchi, 2022, Japan	Retrospective cohort study	1,066 patients (934 ES, 132 EF)	ICU	Machine learning models (Efficient Net-Based Model; LightGBM)	Extubation failure (reintubation or death within 48h)	AUC for Efficient Net-Based Model (CXR only) = 0.55; AUC for LightGBM (RSBI only) = 0.56; AUC for LightGBM (RSBI + CXR) = 0.56; AUC for LightGBM (RSBI + other variables) = 0.70; AUC for LightGBM (RSBI + CXR + other variables) = 0.71	10.1097/CCE.0000000000000718
Gandia, 1992, Spain	Prospective observational study	40 SBT trials (28 SBT success, 12 SBT failures)	ICU	Inspiratory airway occlusion pressure at 0.1 sec. (P01) to	SBT failure	Diagnostic accuracy of P0.1/MIP = 0.875; Diagnostic accuracy of IEQ = 0.875; Diagnostic accuracy of RSBI = 0.825;	10.1007/BF01694360

				maximum inspiratory pressure ratio (P01/MIP), inspiratory effort quotient (IEQ), RSBI (F/Vt)		Diagnostic accuracy of RR = 0.80; Diagnostic accuracy of MIP = 0.80; Diagnostic accuracy of P0.1 = 0.65; Diagnostic accuracy of Ve = 0.675; Diagnostic accuracy of Vt = 0.70	
Genty, 2022, France	Prospective observational study	Derivation cohort: 50 patients (40 ES, 10 EF) after cardiothoracic surgery. Validation cohort: 39 patients (28 ES, 11 EF) after cardiothoracic surgery	Cardiothoracic ICU	Diaphragm ultrasound (diaphragm thickening fraction)	Weaning failure (SBT failure or extubation failure [reintubation within 48h])	AUC for DTFmax = 0.99; AUC for ΔDTFmax = 0.81	10.4187/respcare.09476
Ghosh, 2018, India	Prospective observational study	201 patients (48 EF)	Interdisciplinary ICU	Cumulative fluid balance, HR	Extubation failure (reintubation within 72h)	AUC for fluid balance = 0.6; HR OR = 1.024	10.4103/ijccm.IJCCM_216_18
Gobert, 2017, France	Prospective observational study	92 patients (11 EF)	Medical ICU	CPF (Cough Peak Flow), Vt (tidal volume in the preceding inspiration), pH before extubation	Extubation failure (reintubation or death within 48h)	AUC for CPF = 0.61; AUC for Vt = 0.64; no statistical differences in pH	10.4187/respcare.05460
Gonzalez-Aguirre, 2019, Mexico	Prospective observational study	82 patients (24 EF)	Medical and surgical ICU	B-Lines in LUS, DSF (Diaphragmatic Shortening Fraction), NUTRIC Score, Albumin, Accumulative Balance, RSBI	Extubation failure (reintubation within 48h)	AUC for Albumin = 0.643; AUC for DSF = 0.664; AUC for accumulative balance = 0.691; AUC for B-Lines = 0.819; AUC for Nutric score = 0.655; AUC for RSBI = 0.570	10.1016/j.arbres.2018.09.015
Haaksma, 2021, Netherlands	Prospective observational study	83 patients (15 EF)	ICU	DTF, B-Lines (in Lung Ultrasound BLUE protocol), Left Ventricular Function, Holistic US assessment (incl. Heart, lung and diaphragmatic function), RR, P/F ratio, leukocytes, CRP, Hb, Creatinine	Extubation failure (reintubation within 48h)	AUC for DTF = 0.61; AUC for B-lines = 0.63; AUC for left ventricular function = 0.54; AUC for holistic US assessment = 0.54; RR OR = 1.07 (n.s.); P/F ratio OR = 1.14 (n.s.); CRP OR = 1.00; Leukocytes OR = 1.06 (n.s.); Hb OR = 1.27 (n.s.); Creatinine OR = 1.00 (n.s.)	10.4187/respcare.08679
Hernandez, 2007, Spain	Prospective observational study	93 patients (19 EF)	Medical-surgical ICU	RT50%^Ve (Recovery Time needed to reduce minute ventilation to half the difference between the minute ventilation measured at the end of a successful spontaneous breathing	Extubation failure (reintubation within 48h)	AUC for RT Ve = 0.73; AUC for RT50%ΔVe = 0.75; AUC for combined model = 0.89	10.1378/chest.06-2137

				trial and basal minute ventilation), basal Ve, RT Ve (Recovery Time Ve)			
Hirolli, 2023, India	Prospective observational cohort study	43 neurointensive patients (15 EF)	Neurological ICU	RIS-i (respiratory insufficiency scale–intubated), VISAGE (visual pursuit, swallowing, age, Glasgow Coma Scale for extubation), Ultrasound: C-PEFR (cough peak expiratory flow rate), DE, DTF	Extubation failure (reintubation within 48h)	AUC for RIS-i = 0.963; VISAGE OR = 0.906 (n.s.); C-PEFR OR = 0.981 (n.s.); DE-Tidal OR = 0.277 (n.s.); DE-MAX OR = 0.788 (n.s.); DE-TF OR = 1.004 (n.s.); C-PV OR = 0.838 (n.s.)	10.1007/s12028-023-01695-4
Houze, 2020, France	Prospective observational multicenter study	159 patients (7 EF)	8 ICUs	Global swallowing pattern assessment (9 Items 1 point each: Salivary stasis, holding the head, opening mouth, pursing lips, clenching teeth, sticking out tongue, gag reflex right, gag reflex left, swallowing function)	Extubation failure (reintubation within 72h related to excessive upper airway secretions or aspiration)	Only right and left gag reflexes were associatied with reintubation; right gag reflex OR = 0.12; left gag reflex OR = 0.13	10.4187/respcare.07025
Hsieh, 2018, Taiwan	Retrospective study	3,602 patients (3,417 ES, 185 EF)	8 ICUs	Artificial neural network (ANN); TISS Scale (Therapeutic Intervention Scoring System); RSBI; MEP; HR, P/F ratio	Extubation failure (reintubation or death within 72h)	TISS Scale OR = 1.813; RSBI OR = 2.003; MEP OR = 0.610; HR OR = 1.705; P/F OR = 0.529; AUC for TISS = 0.58; AUC for MEP = 0.58; AUC for RSBI = 0.54; AUC for ANN = 0.85	10.3390/jcm7090240
Huang, 2023, China	Prospective observational multicenter study	88 patients (38 WF)	ICUs	Diaphragmatic excursion and velocity measured via Ultrasonic Speckle Tracking or Manually	Extubation failure (reintubation within 48h)	AUC for DE = 0.659	10.1186/s13054-022-04288-3
Huang, 2014, Taiwan	Prospective observational study	77 patients (13 EF)	ICU	HRV (Heart rate variability)	Extubation failure (reintubation within 72h)	Δln VLF OR = 3.9; Δln TP OR = 6.6	10.1186/cc13705
Huaringa, 2012, USA	Prospective observational study	59 patients (41 WS, 18 WF)	ICU	Weaning index (RSBI x EI x VDI); Elastance index (PIP/NIF); Ventilatory demand index (VDI)	Extubation failure (reintubation within 24h)	AUC for EI = 0.86; AUC for VDI = 0.52; AUC for WI = 0.95	10.1177/0885066612463681

Huo, 2021, China	Prospective observational study	40 patients (15 EF)	ICU	WI (Weaning Index: RSBI × EI × VDI (elasticity index (EI) = peak inspiratory pressure (PIP)/MIP; ventilation demand index (VDI) = MV/10.), RSBI; Ve; MIP	Extubation failure (reintubation within 48h)	A/C group: AUC for WI = 0.917; AUC for RSBI = 0.84; AUC for PIP/MIP = 0.673; AUC for MIP = 0.554; AUC for Ve = 0.66. PSV group: AUC for WI = 0.923; AUC for RSBI = 0.63.	10.21037/apm-21-105
Ibrahim, 2018, Egypt	Prospective observational study	80 patients (43 ES, 37 EF)	Trauma ICU	APACHE II, SCSS (Semi-quantitative cough strength score), GCS, RSBI, P/F ratio	Extubation failure (reintubation within 72h)	APACHE II OR = 0.83; SCSS OR = 2.29; GCS OR = 1.85; RSBI OR = 1.01; P/F OR = 1	10.1007/s12028-018-0539-3
Karthika, 2023, Saudi Arabia, India	Prospective observational study	160 patients (47 WF, 34 EF)	ICUs	GCS, HR, RR, VT, Hb, RSBI 5-120 (rate of change of RSBI from minute 5 to 120 in %)	Extubation failure (reintubation within 24h)	AUC for RSBI 5-120 = 0.933; AUC for RSBI 120 = 0.899; Values of other parameters are not reported.	10.1155/2023/9141441
Kaur, India	Prospective observational study	50 patients (31 ES, 15 SBT Failure, 4 EF)	ICU	RSBI, P0.1s, NIF (maximum inspiratory pressure against occluded airway), DTF (Diaphragmatic thickening fraction), DE (Diaphragmatic excursion)	Weaning failure (SBT failure or extubation failure [reintubation within 48h])	AUC results are not reported; RSBI is followed by NIF, DTF, P0.1; DE showed smallest AUC.	10.4103/ija.ija_312_22
Khamiees, 2001, USA	Prospective observational study	91 patients (100 extubations, 18 EF)	Medical-cardiac ICUs	WCT (white card test), Cough strength (Scale of 0-5), magnitude of endotracheal secretions (none, mild, moderate, abundant), RSBI, P/F ratio, Hb	Extubation failure (reintubation within 72h)	RSBI Rel. R = 1.0 (n.s.); P/F ratio aRel. R = 0.5 (n.s.); Hb Rel. R = 5.3; Secretions Rel. R = 8.7; Suctioning Rel. R = 16.0; WCT Rel R = 3.0; Cough strength Rel. R = 4.0	10.1378/chest.120.4.1262
Kim, 2011, South Korea	Prospective observational study	82 patients (54 PWF)	Medical ICU	RSBI, DE	Extubation failure (reintubation within 48h)	AUC for RSBI = 0.58; AUC for DE1 = 0.61; AUC for DE2 = 0.68	10.1097/CCM.0b013e3182266408
Ko, 2009, USA	Retrospective case series	62 neurocritical patients (51 ES, 11 EF)	Neurocritical care unit	RSBI, P/F ratio, Vt, Minute volume, negative inspiratory force (NIF), SBT on extubation day	Extubation failure (reintubation within 48h)	All parameters were inaccuracte and non-significant predictors for weaning outcome in neurocritical patients.	10.1007/s12028-008-9181-9
Konomi, 2016, Greece	Prospective observational study	42 patients (27 WS, 7 SBT failure, 8 EF)	Multidisciplinary ICU	RSBI, echocardiography, baseline BNP, fluid balance	Weaning failure (SBT failure or extubation failure [reintubation within 48h])	RSBI OR = 1.05; Echocardiography OR = 11.23; BNP OR = 1.0 (n.s.); Fluid balance OR = 1.0 (n.s.)	10.1177/0310057X1604400408

Krieger, 1997, USA	Prospective observational study	49 patients aged 70 or older (38 WS, 11 WF)	Medical ICU	RSBI	Extubation failure (reintubation within 48h)	AUC for RSBI = 0.93	https://doi.org/10.1378/chest.112.4.1029
Kuo, 2006, Taiwan	Prospective observational study	172 patients (106 WS, 54 SBT failure, 12 EF)	Medical ICU, surgical ICU	RSBI, Respiratory rate, Heart rate	Weaning failure (SBT failure or extubation failure [reintubation within 48h])	AUC for RSBI (SBT onset) = 0.59; AUC for RSBI (SBT termination) = 0.77; AUC for RR = 0.70; AUC for HR = 0.58	https://doi.org/10.1016/s0929-6646(09)60135-2
Kutchak, 2015, Brazil	Prospective, observational cross-sectional study	135 neurological patients (90 ES, 45 EF)	ICU	Reflex cough peak expiratory flow (PEF), GCS	Extubation failure (reintubation within 48h)	AUC for PEF = 0.81; AUC for GCS = 0.57	10.1590/S1806-37132015000004453
Kutchak, 2017, Brazil	Prospective observational cohort study	132 patients (90 ES, 42 EF)	ICU	Motor response (grasp and release examiners' hand), tongue protrusion test (sticking out tongue on command)	Extubation failure (reintubation within 48h)	Motor response Rel. R = 1.57; Tongue protrusion test Rel. R = 6.84	10.1590/S1806-37562016000000155
Laguado-Nieto, 2023, Colombia	Prospective cross-sectional study	61 patients (51 ES, 10 ES)	ICU	Diaphragmatic ultrasound (thickening fraction, movement, excursion)	Weaning failure (SBT failure or extubation failure [reintubation within 48h])	Single parameters were moderate or inaccurate predictors; AUC for combined variables in an US model = 0.8794	10.1177/11795484231165940
Lai, 2016, Taiwan	Retrospective data analysis	6,583 patients (6,180 ES, 403 EF)	ICU	APACHE II score, GCS, RSBI, MIP, MEP, Cuff leak test	Extubation failure (reintubation within 48h)	AUC for MEP = 0.60; AUC for RSBI = 0.59; APACHE aOR = 1.00 (n.s.); GCS aOR = 1.00 (n.s.); MIP aOR = 1.00 (n.s.); Cuff leak test aOR = 1.00 (n.s.)	10.1097/MD.0000000000004852
Lara, 2013, Brazil	Prospective observational study	101 CABG patients (89 WS, 12 WF)	ICU	B-type natriuretic peptide (BNP) before and after SBT	Weaning failure (SBT failure or extubation failure [reintubation within 48h])	AUC for BNP (end of SBT) = 0.91	10.6061/clinics/2013(01)oa05
Liu, 2023, China	Retrospective observational study	227 patients (127 ES, 100 EF)	ICU	SOFA, HR, RR, baseline cholinesterase, PaO2, APACHE II	Extubation failure (reintubation within 48h)	SOFA OR = 1.154 (n.s.); HR OR = 1.007 (n.s.); RR OR = 1.012 (n.s.); cholinesterase OR = 1.00 (n.s.); PaO2 OR = 0.989; APACHE II OR = 1.045 (n.s.)	10.3389/fmed.2023.1175089
Liu, 2010, China	Prospective observational study	91 patients (73 ES, 18 EF)	Medical ICU	RSBI, P0.1, P0.1*RSBI	Extubation failure (reintubation within 48h)	AUC for RSBI (1 min) = 0.70; AUC for RSBI (30 min) = 0.82; AUC for RSBI (60 min) = 0.79; AUC for P0.1 (1 min) = 0.79; AUC for P0.1 (30 min) = 0.86); AUC for P0.1 (60 min) = 0.81; AUC for P0.1xRSBI (1 min) = 0.79; AUC for P0.1xRSBI (30 min) = 0.89; AUC for P0.1xRSBI (60 min) = 0.84; AUC for ΔRSBI (30 min) = 0.76; AUC for ΔRSBI (60 min) = 0.75	10.1213/ANE.0b013e3181f4e82e
Louvaris, 2023, Belgium	Prospective observational study	24 patients (16 SBT success, 8 SBT failure)	Medical ICU	Cerebral cortex perfusion, oxygen	SBT failure	AUC for cerebral cortex perfusion = 0.79; oxygen delivery and saturation did not significantly differ.	10.1007/s12028-022-01641-w

				delivery, oxygen saturation			
Ma, 2013, China	Prospective observational study	29 patients (22 WS, 2 SBT failure, 5 EF)	Department of Intensive Care Medicine	NT-proBNP	Weaning failure (SBT failure or extubation failure [reintubation within 48h])	AUC for NT-proBNP = 0.76	10.1177/0300060513490085
Maezawa, 2021, Japan	Retrospective observational study	161 patients (149 ES, 12 EF)	ICU	Fluid balance, RSBI, APACHE II	Extubation failure (reintubation within 72h)	AUC for fluid balance (24h) = 0.771; AUC for fluid balance (48h) = 0.492; AUC for fluid balance (cumulative) = 0.455; AUC for RSBI = 0.547; AUC for APACHE II = 0.765	10.1177/0885066619887694
Maraghi, 2014, Egypt	Prospective observational study	40 patients (25 ES, 8 SBT failure, 7 EF)	Critical care department	Change in BNP	Extubation failure (reintubation within 48h)	AUC for BNP = 0.96	10.1016/j.ejcdt.2014.04.003
Martinez, 2003, USA	Prospective observational study	69 patients (59 ES, 10 EF)	Medical/surgical ICU	Minute ventilation at baseline, post trial and recovery time; RSBI post trial; PaCO2	Extubation failure (reintubation within 7 days)	AUC for Ve (baseline) = 0.53; AUC for Ve (post trial) = 0.62; AUC for Ve (recovery time) = 0.85; AUC for RSBI = 0.57; AUC for PaCO2 = 0.55	10.1378/chest.123.4.1214
Mekontso-Dessap, 2006, France	Prospective observational study	102 patients (60 ES, 5 EF, 37 SBT failure)	ICU	Dynamic compliance, pressure frequency product (PFP), BNP	Extubation failure (reintubation within 48h)	AUC for BNP = 0.89; Cdyn OR = 0.99 (n.s.); PFP OR = 1.09	10.1007/s00134-006-0339-7
Mohsenifar, 1993, USA	Prospective observational cohort study	29 patients (18 WS, 11 WF)	Respiratory ICU	Gastric intramural pH, Vt, RR, Negative inspiratory pressure, RSBI	Extubation failure (reintubation within 24h)	Sensitivity, specificity, PPV and NPV for pHi = 1.0; 1.0; 1.0; 1.0), Vt = 1.0; 0.18; 0.66; 1.0; RR = 1.0; 0.27; 0.69; 1.0; NIF = 1.0; 0.09; 0.64; 1.0; RSBI = 1.0; 0.27; 0.69; 1.0	10.7326/0003-4819-119-8-199310150-00004
Mokhlesi, 2007, USA	Prospective observational study	122 patients (106 ES, 16 EF)	Medical ICU, surgical ICU	Hypercapnia pre-extubation, GCS score, Secretion	Extubation failure (reintubation within 48h)	Hypercapnia OR = 13; GCS OR = 13; Secretion OR = 12; AUC for model 1 (hypercapnia, secretions and/or GCS) = 0.87; AUC for model 2 (hypercapnia, secretions) = 0.83; AUC for model 3 (secretions, GCS) = 0.81; AUC for model 4 (hypercapnia, GCS) = 0.77	PubMed ID 18028561
Moon, 2014, South Korea	Retrospective observational study	64 poisoned patients (61 successful weaning episodes, 9 failed weaning episodes)	ICU	Muscle acetylcholinesterase (AChE)	Extubation failure (reintubation within 72h)	AUC for RBC AChE activity at presentation = 0.812	10.4187/respcare.02916
Moon, 2021, South Korea	Prospective observational study	40 patients (27 ES, 9 SBT failure, 4 EF)	Medical ICU	Electrical impedance tomography (Temporal skew of lung aeration = TSA; change in inhomogeneity index (= ΔGI; uneven ventilation	Weaning failure (SBT failure or extubation failure [reintubation within 48h])	AUC for TSA = 0.94; AUC for RSBI = 0.78; AUC for ΔEELI; AUC for ΔGI = 0.81	10.1016/j.jcrc.2021.06.010

				within lung); change in end-expiratory lung impedance = ΔEELI); RSBI			
Moschietto, 2012, France	Prospective observational study	68 patients (48 ES, 16 SBT failure, 4 EF)	Medical ICU	RSBI, bedside echocardiography	SBT failure, extubation failure (reintubation within 48h)	AUC for E/Ea (baseline) = 0.75; AUC for E/Ea (SBT) = 0.86; AUC for RSBI = 0.77; AUC for E (baseline) = 0.6; AUC for DTE = 0.67; AUC for Ea (baseline) = 0.69	10.1186/cc11339
Mostafa, 2022, Egypt	Prospective observational study	39 patients (17 WS, 22 WF)	Surgical ICU	Heart ultrasound using transesophageal doppler	Weaning failure (SBT failure or extubation failure [reintubation within 48h])	Various AUC results; Delta change in peak velocity and cardiac output were predictive for weaning outcome in cardiac patients, but not in non-cardiac patients; central venous oxygen saturation and corrected flow time were no significant predictors of weaning outcome.	10.1053/j.jvca.2022.01.010
Norisue, 2021, Japan	Prospective observational cohort study	252 patients (240 ES, 12 EF)	Medical-surgical ICU	Diaphragm movement during cough (passive cephalic excursion of the diaphragm (PCED), diaphragm peak velocity, cough peak flow (CPF))	Extubation failure (reintubation within 72h)	AUC for PCED = 0.791; AUC for peak velocity = 0.587; AUC for CPF = 0.765	10.4187/respcare.09007
Osman, 2017, Egypt	Prospective observational study	68 patients (50 ES, 18 EF)	ICUs	DE, DTF, Lung ultrasound score	Extubation failure (reintubation within 48h)	AUC for DE = 0.833; AUC for DTF = 0.889; AUC for LUS = 0.942	10.1016/j.ejrnm.2017.01.005
Otaguro, 2021, Japan	Retrospective observational study	117 patients (104 ES, 13 EF)	ICU	Machine learning algorithms (Random Forest, XGBoost, LightGBM)	Extubation failure (reintubation within 72h)	AUC for Random Forest = 0.936; AUC for XGBoost = 0.948; AUC for LightGBM = 0.950	10.1272/jnms.JNMS.2021_88-508
Pan, 2023, China	Prospective observational study	163 patients (151 ES, 12 EF)	ICU	Variability of PIP, RR, Vt, Ve, RSBI, and MP as single parameters; Combined parameters in different machine learning models (XGBoost)	Extubation failure (reintubation within 48h)	Various AUC analysis for single parameters; AUC for XGBoost models for groups between 0.781 and 0.902	10.1016/j.compbiomed.2022.106459
Papanikolaou, 2011, Greece	Prospective observational study	50 patients (22 WS, 23 SBT failure, 5 EF)	ICU	APACHE II, RSBI, Pressure-frequency product (PFP), Echocardiography	Weaning failure (SBT failure or extubation failure [reintubation within 48h])	AUC for APACHE II = 0.67; AUC for RSBI = 0.75; AUC for PFP = 0.76; AUC for lateral Em = 0.80; AUC for averaged Em = 0.78; AUC for septal Sm = 0.74; AUC for lateral E/Em = 0.86; AUC for septal E/Em = 0.81; AUC for averaged E/Em = 0.86; AUC for E/Vp = 0.74	10.1007/s00134-011-2368-0

Park, 2023, South Korea	Retrospective data analysis	138 patients (103 WS, 35 WF)	Medical ICU	Weaning prediction model based on a convolutional neural network (CNN), RSBI	Extubation failure (reintubation within 48h)	AUC for CNN = 0.912; Precision for CNN = 0767; AUC for RSBI = 0.558; Precision for RSBI = 0.522	10.3390/bioengineering10101163
Piriyapatsom, 2016, USA	Prospective observational study	764 patients (699 ES , 65 EF)	2 Surgical ICUs	Muscle strength, Hemoglobin, blood urea nitrogen level (BUN)	Extubation failure (reintubation within 72h)	BUN OR = 3.66; Hb OR = 2.10; Muscle strength OR = 2.03	10.4187/respcare.04269
Ramaswamy, 2023, India	Prospective observational study	60 patients (41 WS, 11 SBT failure, 8 EF)	ICU	Parasternal intercostal muscal ultrasound (muscle thickness inspiration & expiration; thickening fraction)	Weaning failure (SBT failure or extubation failure [reintubation within 48h])	AUC for Parasternal intercostal muscle thickness fraction (PICTF%) = 0.875	10.5005/jp-journals-10071-24548
Raurich, 2008, Spain	Prospective observational study	103 patients (56 WS, 36 SBT failure, 11 EF)	2 Medical-surgical ICUs	Hypercapnia-test P.01/baseline P0.1; ΔVe/ΔPaCO2; ΔP0.1/ΔPaCO2	Weaning failure (SBT failure or extubation failure [reintubation within 48h])	AUC for hypercapnia test = 0.80; AUC for ΔVe/ΔPaCO2 = 0.86; AUC for ΔP0.1/ΔPaCO2 = 0.82	No DOI; PMID: 18655738
Raurich, 2009, Spain	Prospective observational study	44 COPD patients (19 WS, 25 WF)	2 Medical ICUs	PaCO2 (Baseline, after hypercapnia test); ΔP0.1/ΔPaCO2; ΔVe/ΔPaCO2; ΔP0.1/PaCO2; ΔVe/PaCO2	Weaning failure (SBT failure or extubation failure [reintubation within 48h])	AUC for baseline PaCO2 = 0.81; AUC for hypercapnia test = 0.76; AUC for ΔP0.1/ΔPaCO2 = 0.74; AUC for ΔVe/ΔPaCO2 = 0.76; AUC for ΔP0.1/PaCO2 = 0.76; AUC for ΔVe/PaCO2 = 0.81	10.1177/0310057X0903700507
Rizzo, 2021, USA	Retrospective data analysis	583 burn patients (511 WS, 72 WF)	Burn ICU	Total body surface area (TBSA) burned, respiratory rate, pH, heart rate	Extubation failure (reintubation within 72h)	Heart rate was an independent predictor of extubation outcome (log. reg., no values reported).	10.1093/jbcr/iraa162
Saeed, 2016, Egypt	Prospective observational study	30 patients (21 ES. 9 EF)	Respiratory ICU	Diaphragmatic displacement (DD); RSBI; VT; MIP; Ve; P/F ratio	Extubation failure (reintubation within 48h)	AUC for DD = 0.895; AUC for RSBI = 0.85; AUC for VT = 0.846; AUC for MIP = 0.724; AUC for Ve = 0.673; AUC for P/F = 0.528	10.4103/1687-8426.184363
Sahu, 2022, India	Prospective observational pilot study	27 cirrhotic patients (18 WS, 9 WF)	Liver ICU	Integrative weaning index	SBT failure	AUC for IWI = 0.994	10.5152/TJAR.2021.1057
Salam, 2004, USA	Prospective observational study	88 patients (74 ES, 14 EF)	Medical-cardiac ICU	Cough peak flow, Following commands, White card test, RSBI	Extubation failure (reintubation within 72h)	CPF Risk ratio = 4.8; Secretions Risk ratio = 3.0; Commands Risk ratio = 4.3; WCT Risk ratio = 2.3; RSBI Risk ratio = 1.9	10.1007/s00134-004-2231-7
Santos Lima, 2013, Brazil	Prospective observational study	166 patients (127 WS, 29 WF, 10 EF)	ICU	Respiratory rate	Weaning failure (SBT failure or extubation failure [reintubation within 48h])	RR Sensitivity = 1.0; RR specificity = 0.85; RR NPV = 1.0; RR PPV = 0.60	10.1016/S0034-7094(13)70194-6
Saugel, 2012, Germany	Retrospective data analysis	61 patients (54 ES, 7 EF)	Medical ICU	Serium anion gap, P/F ratio	Extubation failure (reintubation within 48h)	AUC for Serium anion gap = 0.835; AUC for P/F ratio = 0.733	10.1016/j.jcrc.2012.01.010
Savi, 2012, Brazil	Prospective observatonal study	500 patients (386 ES, 114 EF)	3 Medical-surgical ICUs	P/F ratio, CROP, PaCO2, PaO2, RR, Vt, RSBI, MIP	Extubation failure (reintubation within 48h)	None of the parameters was predictive for weaning outcome.	10.1016/j.jcrc.2011.07.079

Schönhofer, 2004, Germany	Prospective observational study	246 patiens (146 WS, 100 WF)	Respiratory ICU	APACHE II	Weaning failure (reintubation within 24h, death within 72h)	AUC for APACHE II = 0.638; AUC for modified APACHE II = 0.712	10.1017/s0265021504007100
Segal, 2010, USA	Prospective observational study	72 patients (63 ES, 9 EF)	Medical/surgical ICU	Change in RSBI during SBT; RR; VT	Extubation failure (reintubation within 48h)	AUC for initial RSBI = 0.43; AUC for 30-min RSBI = 0.83; AUC for max. change in RSBI = 0.93); AUC for RR = 0.76; AUC for VT = 0.78	10.1007/s00134-009-1735-6
Segura, 2011, Colombia	Retrospective data analysis	332 patients (280 WS, 52 WF)	ICU	RSBI	Extubation failure (reintubation within 48h)	AUC for RSBI = 0.545	10.25100/cm.v42i4.946
Seymour, 2008, USA	Prospective observational cohort study	88 patients (66 WS, 22 WF)	Surgical ICU, Medical ICU	Minute ventilation recovery time	Extubation failure (reintubation within 7 days)	AUC for Ve recovery time = 0.79	10.1177/0885066607310302
Shin, 2017, South Korea	Retrospective data analysis	127 patients under prolonged MV (41 WS, 86 WF)	Medical ICU	SOFA Score (on day 21 of MV)	Weaning success/failure (extubation/decannulation and no reintubation within 7 days)	AUC for SOFA = 0.77	10.21037/jtd.2017.01.14
Smailes, 2013, UK	Prospective observational study	125 patients (108 ES, 17 EF)	Burn ICU	Cough peak flow, secretion score, CPF/Secretion Model	Extubation failure (reintubation within 48h)	CPF OR = 1.06; Secretion score OR = 0.005; AUC for CPF/Secretion Model = 0.96	10.1016/j.burns.2012.09.028
Smina, 2003, USA	Prospective observational study	95 patients with 115 extubation (102 ES, 13 EF)	Medical-cardiac ICUs	Cough peak expiratory flow (PEF); APACHE II; RSBI; Hemoglobin	Extubation failure (reintubation within 72h)	AUC for cough PEF = 0.70; Rel. R APACHE II = 5.2; Rel. R RSBI = 5.2; Rel. R Hb = 5.1	10.1378/chest.124.1.262
Soliman, 2019, Egypt	Prospective observational study	100 patients (80 WS, 20 WF)	Department of critical care medicine	DTF, LUS, RSBI	Weaning failure (SBT failure or extubation failure [reintubation within 48h])	DTF was a good predictor for weaning failure; LUS and RSBI were moderate predictors. No AUC values are reported.	10.3889/oamjms.2019.277
Takaki, 2015, Malaysia	Prospective observational study	96 patients (84 WS, 12 WF)	2 ICUs	RSBI, RSBI modified with actual body weight, predicted body weight, ideal body weight, BMI, and body surface area	Extubation failure (reintubation within 48h)	AUC for RSBI = 0.95; AUC for RSBI with actual body weight = 0.98; AUC for RSBI with BMI = 0.98; AUC for RSBI with predicted body weight = 0.94; AUC for RSBI with ideal body weight = 0.94; AUC for RSBI with body surface area = 0.98	10.1053/j.jvca.2014.06.022
Teixeira, 2010, Brazil	Prospective observational multicentric study	73 patients (42 WS, 31 WF)	3 ICUs	$ScvO_2$	Extubation failure (reintubation within 48h)	AUC for $ScvO_2$ = 0.87	10.1097/CCM.0b013e3181bc81ec
Thille, 2015, France	Prospective observational study	225 patients (191 WS, 31 WF)	Medical ICU	Secretion, cough strength	Extubation failure (reintubation within 7 days); Extubation failure (reintubation within 72h)	Secretions aOR = 3.32 (reintubation 72h); Secretions aOR = n.s. (reintubation 7d); Cough strength aOR = 5.03 (reintubation 72h); Cough strength aOR = 5.09 (reintubation 7d)	10.1097/CCM.0000000000000748
Tongyoo, 2019, Thailand	Prospective observational cohort study	52 patients (38 WS, 14 WF)	Medical ICU	Echochardiography, BMI	Extubation failure (reintubation within 48h)	AUC for BMI = 0.68; AUC for Peak A wave = 0.35; AUC for E/Ea = 0.70; AUC for E/A = 0.65; AUC for IVC maximum diameter = 0.71	10.1111/echo.14306

Upadya, 2005, USA	Prospective observational study	87 patients (39 WS, 48 WF)	Medical ICU	RSBI, fluid balance	Weaning failure (SBT failure or extubation failure [reintubation within 72h])	24h fluid balance OR = 2.9; cumulative negative fluid balance OR = 3.4; RSBI value not reported.	10.1007/s00134-005-2801-3
Uusaro, 2000, Canada	Prospective observational study	68 patients (51 WS, 17 WF)	ICU	RR, Vt, P0.1, gastric-arterial paC02 (ΔPg-aCO2), RSBI, Minute ventilation	Extubation failure (reintubation within 24h)	AUC for Vt = 0.63; AUC for RSBI = 0.68; AUC for P0.1 = 0.63; AUC for ΔPg-aCO2 = 0.54	10.1097/00003246-200007000-00022
Vassilakopoulos, 1998, Greece	Prospective observational study	30 patients (all initially failed and later succeded in weaning)	ICU	Tension-Time-Index, RSBI	SBT failure	TTI aOR = 10.28; RSBI aOR = 3.19	10.1164/ajrccm.158.2.9710084
Verona, 2015, Brazil	Prospective observational study	34 patients (21 WS, 13 WF)	Mixed ICU	Malondialdehyde (MDA), vitamine C, nitric oxid concentration	SBT failure	None of the parameters differed significantly.	10.1111/jcmm.12475
Vetrugno, 2022, Italy	Prospective observational multicenter study	57 patients (32 WS, 25 WF)	6 ICUs	Diaphragm ultrasound (diaphragm thickening fraction)	Weaning failure (SBT failure or extubation failure [reintubation within 48h])	No significant difference in DTF values of failure and success groups.	10.1186/s12931-022-02138-y
Vidotto, 2008, Brazil	Prospective observational cohort study	92 patients (77 WS, 15 WF)	Neurosurgical ICU	RSBI, RR	Extubation failure (reintubation within 48h)	AUC for RSBI = 0.69; AUC for RR = 0.74	https://doi.org/10.1007/s12028-008-9059-x
Wang D, 2023, China	Prospective observational study	60 patients (50 WS, 10 WF)	Medical ICU	Pendelluft (assessed by electrical impedance tomography)	SBT failure	AUC for ventral pendelluft = 0.76; AUC for total pendelluft = 0.75	https://doi.org/10.3389/fphys.2023.1113379
Wang G, 2021, China	Prospective observational case-control study	53 patients (41 WS, 12 WF)	Department of Intensive Care Medicine	Electrical impedance tomography	Weaning failure (SBT failure or extubation failure [reintubation within 48h])	AUC for global impendance of pre-SBT = 0.686; AUC for region of interest 2 of SBT = 0.768	https://doi.org/10.2147/ijgm.s331772
Weber, 2021, Israel	Retrospective cohort study	329 patients	Medical ICU	Alanine Aminotransferase (ALT)	Extubation failure (reintubation within 48h); Extubation failure (reintubation within 7 days)	AUC for ALT = 0.62 (reintubation 48h); AUC for ALT = 0.61 (reintubation 7 days)	https://doi.org/10.3390/jcm10153282
Wendell, 2011, USA	Retrospective cohort study	47 patients (37 ES, 10 EF)	ICUs	GCS	Extubation failure (reintubation within 48h)	GCS OR = 23.30 (n.s.)	10.4061/2011/248789
Wu, 2019, Taiwan	Prospective observational study	59 patients with 70 extubations (56 successful, 14 failure)	Medical ICU	Albumin, Hemoglobin, GCS, RSBI	Extubation failure (reintubation within 48h)	AUC for Albumin = 0.73; AUC for Hb = 0.71; AUC for GCS = 0.75; AUC for RSBI = 0.60	10.1177/0885066617706688
Xiao, 2018, China	Prospective observational study	139 patients (117 ES, 22 EF)	Respiratory ICU	RSBI; APACHE II; SBT attempts; Secretions; CPF; Hemoglobin; pH; PaCO2; P/F ratio;	Extubation failure (reintubation within 72h)	RSBI OR = 1.009 (n.s.); APACHE II OR = 1.174; SBT attempts OR = 1.446 (Mult. Log. Reg.); Secretions OR = 1.002 (n.s.); CPF OR = 0.975 (Mult. Log. Reg.); Hb OR = 0.9871 (n.s.); pH OR = 0.0000 (n.s.);	10.1111/crj.12657

				Prealbumin; Albumin; Total proteins		PaCO2 OR = 1.038; Horovitz OR = 0.995 (n.s.); Prealbumin OR = 0.991 (n.s.); Albumin OR = 0.847 (Mult. log. reg.); Total proteins OR = 0.953 (n.s.)	
Xu SS, 2023, China	Prospective observational diagnostic study	226 neurosurgical patients (183 ES, 43 EF)	2 ICUs	STAGE Score (Swallowing, tongue protrusion, spontaneous cough, suctioning cough, motor response in GCS)	Extubation failure (reintubation within 72h)	Swallowing OR = 2.86; Tongue protrusion OR = 1.84 (n.s.); Cough OR = 2.03 (n.s.); Suctioning cough OR = 2.83; Motor response in GCS OR = 1.72 (n.s.)	10.1097/aln.0000000000004721
Xu Q, 2022, China	Retrospective study	96 patients (59 WS, 37 WF)	2 ICUs	Diaphragm ultrasound (DE, DTF, longitudinal strain DLS), RSBI	Extubation failure (reintubation within 48h or death within 7 days)	AUC for RSBI = 0.794; AUC for DLS = 0.794; AUC for DE = 0.728; AUC for DTF = 0.723	10.1186/s12890-022-02260-z
Yang, 1993, USA	Prospective observational study	31 patients (16 SW, 15 SBT failure)	ICU	RR, Vt, RR/Vt, Ve, Pi, MIP, Pi/MIP	Weaning failure (SBT failure or extubation failure [reintubation within 24h])	Sensitivity, specificity, PPV and NPV for RR (0.88; 0.47; 0.64; 0.78), Vt (1.0; 0.67; 0.76; 1.0), RSBI (0.94; 0.73; 0.79; 0.92), Ve (0.81; 0.20; 0.52; 0.50), MIP (1.0; 0.13; 0.55; 1.0), Pi/MIP (0.75; 0.67; 0.71; 0.72), RSBI and Pi/MIP (0.81; 0.93; 0.93; 0.83)	10.1007/bf01694771
Yang, 1991, USA	Prospective observational study	100 patients (60 SW, 40 WF)	Medical ICU	Ve, RR, Vt, Vt/weight, MIP, Dynamic compliance, Static compliance, PaO2/PAO2, RSBI, CROP index	Weaning failure (SBT failure or extubation failure [reintubation within 24h])	AUC for Ve = 0.40; AUC for RR = 0.76; AUC for Vt = 0.87; AUC for Vt/weight = 0.84; AUC for MIP = 0.61; AUC for dynamic compliance = 0.67; AUC for static compliance = 0.68; AUC for PaO2/PAO2 ratio = 0.48; AUC for RSBI = 0.89; AUC for CROP index = 0.78	10.1056/nejm199105233242101
Yu, 2020, China	Retrospective cohort study	125 pneumonia patients (82 WS, 43 WF)	Respiratory ICU	APACHE II, Lung injury score (LIS), GOCA score (gas exchange, organ failure, cause, associated disease), RR, pH, P/F ratio, HCO3, Bilirubin, Glucose, APTT (activated partial thromboplastin time), BNP, PEEP, FiO2	Extubation failure (reintubation within 72h)	AUC for APACHE = 0.644; AUC for blood glucose = 0.585; LIS OR = 1.596 (n.s.); GOCA OR = 1.356; RR OR = 1.072; pH OR = 0.009; P/F ratio OR = 0.995 (n.s.); HCO3 OR = 0.923 (n.s.); Bilirubin OR = 1.035 (n.s.); APTT OR = 1.020 (n.s.); BNP OR = 1.000 (n.s.); PEEP OR = 1.132; FiO2 OR = 1.018	10.1042/bsr20192435
Zeggwagh, 1999, Morocco	Prospective observational study	101 patients (53 development series; 48 validation series)	Medical ICU	RR, RSBI, Vt, VC, Ve, MIP, MEP	Extubation failure (reintubation within 48h)	AUC for RR = 0.708; AUC for Vt = 0.830; AUC for RSBI = 0.809; AUC for VC = 0.812; AUC for Ve = 0.341; AUC for MIP = 0.715; AUC for MEP = 0.726	10.1007/s001340051015
Zeid, 2021, Egypt	Prospective randomized observational study	80 patients (40 CPAP with 11 SBT failure and 10 reintubation; 40 T-piece with 9 SBT	Department of Critical Care Medicine	Focused trans-thoracic echocardiography (left and right ventricle)	Weaning failure (SBT failure or extubation failure [reintubation within 48h])	AUC for E/A CPAP = 0.703; AUC for Septal E/E' CPAP = 0.732; AUC for TAPSE CPAP = 0.456; AUC for IJV distensibilty index = 0.499; AUC for IVC distensibilty index; AUC for E/A T-tube = 0.487; AUC for	10.3889/oamjms.2021.7026

		failure, 9 reintubation)				Septal E/E’ T-tube = 0.826; AUC for TAPSE T-tube = 0.597; AUC for IJV collapsibility index = 0.525; AUC for IVC collapsibility index	
Zhang, 2014, China	RCT	208 patients (168 WS, 40 WF)	Internal ICU, Medical ICU	RSBI	SBT failure	PSV group: AUC for RSBI = 0.747; AUC for ΔRSBI = 0.709; T-piece group: AUC for RSBI = 0.821; AUC for ΔRSBI = 0.738	10.1097/MAJ.0000000000000286
Zhao, 2021, China	Retrospective data analysis	33 COVID-19 patients (15 WS, 18 WF)	ICU	Pplat, Driving pressure; Compliance	Weaning failure (SBT failure or extubation failure [reintubation within 48h])	Pplat OR = 1.81; Driving pressure OR = 0.551; Compliance OR = 0.55	10.3389/fmed.2021.678157
Zheng, 2022, China	Retrospective observational study	261 patients (45 SBT failure, 9 extubation failure)	ICU	Mean platelet volume (MPV), Leukocyte, CRP	Weaning failure (SBT failure or extubation failure [reintubation within 48h])	AUC for MPV = 0.777; AUC for Leukocyte = 0.6; AUC for CRP = 0.627	10.1186/s12871-022-01701-w
Zheng, 2023, China	Retrospective observational cohort study	323 postsurgical patients (31 SBT failure, 14 extubation failure)	ICU	NT-proBNP, RSBI, MV	Weaning failure (SBT failure or extubation failure [reintubation within 48h])	AUC for ΔNTproBNP% = 0.744; AUC for NTproBNP1 = 0.639; AUC for NTproBNP2 = 0.742; AUC for RSBI = 0.651; AUC for MV = 0.552	10.1186/s12871-023-02039-7
